# Discovery and Characterization of a Novel Tomato *mlo* Mutant from an EMS Mutagenized Micro-Tom Population

**DOI:** 10.3390/genes12050719

**Published:** 2021-05-11

**Authors:** Zhe Yan, Michela Appiano, Ageeth van Tuinen, Fien Meijer-Dekens, Danny Schipper, Dongli Gao, Robin Huibers, Richard G. F. Visser, Yuling Bai, Anne-Marie A. Wolters

**Affiliations:** Plant Breeding, Wageningen University & Research, P.O. Box 386, 6700 AJ Wageningen, The Netherlands; zhe.yan@wur.nl (Z.Y.); michela.appiano@gmail.com (M.A.); ageeth.vantuinen@wur.nl (A.v.T.); fien.meijer-dekens@wur.nl (F.M.-D.); danny.schipper@wur.nl (D.S.); gdongli@126.com (D.G.); R.Huibers@enzazaden.nl (R.H.); richard.visser@wur.nl (R.G.F.V.); bai.yuling@wur.nl (Y.B.)

**Keywords:** *Solanum lycopersicum*, Micro-Tom, EMS mutagenesis, powdery mildew, *Oidium neolycopersici*, *SlMLO1*

## Abstract

In tomato (*Solanum lycopersicum*), there are at least three *SlMLO* (*Mildew resistance Locus O*) genes acting as susceptibility genes for the powdery mildew disease caused by *Oidium neolycopersici*, namely *SlMLO1*, *SlMLO5* and *SlMLO8*. Of the three homologs, the *SlMLO1* gene plays a major role since a natural mutant allele called *ol-2* can almost completely prevent fungal penetration by formation of papillae. The *ol-2* allele contains a 19-bp deletion in the coding sequence of the *SlMLO1* gene, resulting in a premature stop codon within the second cytoplasmic loop of the predicted protein. In this study, we have developed a new genetic resource (M200) in the tomato cv. Micro-Tom genetic background by means of ethyl methane sulfonate (EMS) mutagenesis. The mutant M200 containing a novel allele (the *m200* allele) of the tomato *SlMLO1* gene showed profound resistance against powdery mildew with no fungal sporulation. Compared to the coding sequence of the *SlMLO1* gene, the *m200* allele carries a point mutation at T65A. The SNP results in a premature stop codon L22* located in the first transmembrane domain of the complete SlMLO1 protein. The length of the predicted protein is 21 amino acids, while the SlMLO1 full-length protein is 513 amino acids. A high-resolution melting (HRM) marker was developed to distinguish the mutated *m200* allele from the *SlMLO1* allele in backcross populations. The mutant allele conferred recessive resistance that was associated with papillae formation at fungal penetration sites of plant epidermal cells. A comprehensive list of known *mlo* mutations found in natural and artificial mutants is presented, which serves as a particularly valuable resource for powdery mildew resistance breeding.

## 1. Introduction

Tomato (*Solanum lycopersicum* L.) is a model crop species of high economic value with interesting developmental features such as compound leaves, fleshy fruits, and sympodial shoot branching. The amount of information currently available for the domesticated tomato is abundant. Its genome [1], transcriptome (Tomato Functional Genomics Database, http://ted.bti.cornell.edu/) and metabolome [2] are available, as well as functional genomic tools, like RNA interference (RNAi, [3,4,5]), transcription activator-like effector nucleases (TALENs, [6]), and clustered regularly interspaced short palindromic repeats (CRISPR)/Cas9-based directed mutagenesis [7,8]. 

An important aspect of the domesticated tomato is its lack of genetic diversity because of years of selection for a limited set of traits, such as fruit shape and size [9]. However, given the upcoming challenges for agriculture regarding climate change and food safety, it has become a prominent issue to improve tomato also for resistance or tolerance to biotic and abiotic stresses [10]. One way to achieve this goal is to use the diversity present in wild relatives. It has been a general practice in tomato breeding to use wild relatives as a donor for introgression of valuable traits present in tomato varieties. Another way to increase genetic diversity is to introduce new mutations artificially. Chemical and physical mutagenesis are frequently used for this purpose in most of the economically important crop species [11]. Of the chemical mutagens, ethyl methane sulfonate (EMS) is most commonly used. EMS selectively alkylates guanine bases, which, during DNA replication, are preferably coupled with a thymine over a cytosine residue, resulting in a random point mutation. Most of these mutations (70–99%) consist of substitutions from C to T or from G to A (abbreviated as C/G to T/A) [12,13,14]. Five EMS tomato populations were developed during the last years, two of which used the tomato cultivar Micro-Tom (MT) [15,16,17,18,19]. In contrast to most of the cultivated tomatoes, MT is a miniature determinate tomato cultivar (8–10 cm when grown in 14 cm diameter pots) and has a short life cycle (70–90 d from sowing to fruit ripening) [15,20]. MT has been compared to *Arabidopsis* as a model system to carry out molecular research in tomato. The Japanese mutant database, TOMATOMA has become available, together with MT’s genome and a whole-genome resequencing analysis of EMS-MT mutants [21,22]. Collectively, these features make MT a suitable cultivar for large-scale mutagenesis studies. 

Breeders aim at finding and introducing durable resistance in cultivated crops. One way to achieve this consists of using impaired plant susceptibility genes (*S*-genes) [23,24]. The *Mildew resistance locus o* (*MLO*) gene is the best characterized example of *S*-genes in several crops. Natural and EMS-induced loss-of-function mutants of *MLO* were first detected in powdery mildew (PM)-resistant barley. This *mlo*-based resistance has been successfully employed in agriculture for nearly five decades [25,26,27]. 

*MLO* is a member of a medium-sized gene family [28]. The *MLO* genes encode plant transmembrane proteins which typically span across the plasma membrane seven times and end in the cytoplasm with a C-terminal domain. MLO proteins seem to be involved in many biological processes, although their core biochemical function is still unknown. These proteins likely act in signal transduction in a calcium and calmodulin dependent manner [29,30]. In *Arabidopsis* and barley, pleiotropic effects associated with the disruption of *MLO* function consist of aberrant root architecture (*AtMLO4* and *AtMLO11*) [31,32], reduced fertility (*AtMLO7*) [33], induced lesions (*HvMLO1* and *HvMLO3*) [34,35], early leaf senescence (*HvMLO5*) [36], reduced root colonization by mycorrhizal fungi [37], and susceptibility to several hemibiotrophic and necrotrophic pathogens [30,38,39,40,41].

The *MLO* gene conferring PM resistance is highly conserved in plant species and can be tracked back to green algae [42]. Each plant species contains a certain number of *MLO* paralogs. In a given species, identification of the respective *MLO* paralogs that confer PM susceptibility is a prerequisite for the subsequent utilization of *mlo* alleles. Members of clade IV in monocots and V in dicots are described as susceptibility factors towards pathogens causing the PM disease [42,43,44,45]. Functional proteins of these genes are required by adapted PM pathogens to be able to penetrate the cell wall and cause disease [30,46,47]. In tomato, the *SlMLO* gene family comprises 16 homologs, of which four belong to clade V, namely *SlMLO1*, *SlMLO3*, *SlMLO5* and *SlMLO8* [48].

In addition to barley, natural *mlo* mutants of different types (i.e., transposon insertions, single nucleotide polymorphisms, and small indels) have been found in many plant species, including cucumber (*CsaMLO8* [49]), melon (*CmMLO2* [50]), pea (*er-1*, *-2*, *-3*, and *-4* [51], *er-6* [52], and *er-7* [53]), rose (*RhMLO4* [54]), apple (*MdMLO19* [55]), and tobacco (*NtMLO2* [56]). These examples demonstrate that naturally occurring allelic variants represent a rich source for *mlo*-mutants. In addition, loss-of-function mutations have been obtained through targeted genome editing technologies. These include TALEN-induced *Tamlo* triple-mutant lines and CRISPR/Cas9-mediated mutagenesis of *TaMLO-A1* allele in hexaploid wheat [57], CRISPR/Cas9-induced *SlMLO1* mutant in tomato [58], and CRISPR/Cas9-induced *VvMLO3* mutant in grapevine [59]. When looking at all the *mlo* mutant alleles obtained with mutagens, the highest number is found in barley (33 [60]), followed by wheat (16 [61]), pea (3 [62,63]), and petunia (2 [64]). In tomato and several other plant species (apple, melon, pea, tobacco) no pleiotropic effects are described for mutants of *MLO* genes conferring resistance to adapted PM species [30].

A naturally mutated allele of the *SlMLO1* gene Solyc04g049090.3, called *ol-2*, was described in the past years [65,66,67,68]. The *ol-2* variant contains a 19-bp deletion in the coding sequence resulting in a premature stop codon within the second cytoplasmic loop of the predicted protein. This mutation, first identified in *S. lycopersicum* var. *cerasiforme*, when in homozygous state, mediates broad-spectrum resistance to *Oidium neolycopersici*, recently also referred to as *Pseudoidium neolycopersici* (Mycobank database; https://www.mycobank.org/page/Basic%20names%20search). The *ol-2* conferred resistance is characterized by the formation of papillae beneath the fungal appressoria, which can significantly reduce the fungal penetration [69]. In the following years, transgenic RNAi lines were developed to silence simultaneously multiple clade V-*SlMLO* homologs [48,69]. One construct, in particular, was described to silence *SlMLO1*, *SlMLO5* and *SlMLO8*. When *ol-2* plants were compared to plants of the RNAi lines, a higher level of resistance was observed associated with the latter. Because of these results, it was concluded that the three *SlMLO* genes contribute to the tomato susceptibility towards PM, with *SlMLO1* having the major role [48]. 

In the present study, we describe the in-house development of an EMS mutant population of the tomato cultivar MT. The purpose of this EMS population is to select mutants resistant to different tomato pathogens, and to identify the causal mutant S-genes. In this EMS population, a mutant called M200 was uncovered that showed profound resistance to tomato PM. It was shown to be defective in the *SlMLO1* gene. Then, we performed a comparison of the novel allele with the *ol-2* mutation in different genetic backgrounds as well as RNAi lines in which three clade V *SlMLO* homologues are silenced. Results and implications are further presented and discussed in the context of *mlo* mutations occurring in other plant species.

## 2. Materials and Methods

### 2.1. Development of the Micro-Tom EMS Population

Seeds of the tomato cultivar Micro-Tom (MT) were obtained from the Beekenkamp Plants B.V. company (Maasdijk, The Netherlands). First, to determine which concentration of EMS (ethyl methane sulfonate) solution should be used for efficient mutagenesis, a pilot experiment was performed. A batch of approximately 1000 MT seeds (M_0_) was presoaked in distilled water for 8 h and treated overnight with three concentrations of an EMS solution, 0.5% (*v*/*v*), 0.75% (*v*/*v*) and 1% (*v*/*v*), respectively. The obtained M_1_ seeds were then thoroughly washed with distilled water, sown in the greenhouse of Unifarm of Wageningen University and Research, The Netherlands, and grown at a day/night temperature of 21/19 °C and relative humidity of 60% during a 16 h day/8 h night regime. Three-week-old seedlings were transplanted individually to 14 cm pots and grown until 5 to 10 fruits per plant could be harvested. M_2_ seeds were collected from these fruits, surface sterilized in 2% (*v*/*v*) of HCl (Hydrogen chloride) and disinfected in phosphate solution for a minimum of one hour, followed by air drying.

EMS treatment of approximately 1000 MT seeds (M_0_) was repeated four more times (five batches in total). Since several studies showed that the 1% EMS concentration yielded almost two fold more mutations per genome than other concentrations, like 0.5% or 0.75%, without affecting too much the rate of viability [19,21], only the 1% EMS dilution was used for the latter four seed batches.

### 2.2. Powdery Mildew Disease Assays and Quantification of Relative Fungal Biomass

Multiple disease assays were performed to test for PM resistance. These assays involved the screening of approximately 2000 M_1_ plants originating from the first two batches of EMS treated M_0_ seeds, testing segregating families (BC_1_S_1_ families derived from M200 × MT and F_2_ from M200 × MM) for linkage analysis of PM resistance and the *m200* allele, as well as further generations and control genotypes for PM phenotypic evaluation and histological analysis. Four-week-old plants were inoculated with a fresh suspension of *Oidium neolycopersici* (*On*-Wageningen isolate) conidiospores. The *On* isolate was maintained on tomato cv. Moneymaker (MM) as previously described [69]. The suspension was made by rinsing heavily sporulating leaves of the cultivar MM with tap water and adjusting this suspension to a concentration of 2 × 10^4^ spores per milliliter. Ten to fifteen days after inoculation, the plants were visually inspected. To each plant, a score was given based on a disease index (DI) varying from 0 to 3, where 0 indicates that no fungal sporulation is visible and three that fungal colonies cover most of the surface of the inoculated leaves, as in the cv. MM. 

For the quantification of relative PM fungal biomass in infected mutant, RNAi-silenced and control genotypes (see Section 2.3 *Plant Materials*), the third and fourth true leaf of each infected plant were harvested and snap-frozen in liquid nitrogen. The samples were ground in liquid nitrogen with mortar and pestle. Plant and fungal genomic DNAs (gDNA) were isolated using the DNeasy Plant Mini Kit (Qiagen, Hilden, Germany). Isolated DNA was used for qPCR with the primer pairs On_ITS, designed on *O. neolycopersici* internal transcribed spacer (ITS) sequence (GenBank accession number EU047564), and *SlEF1α*, designed on the tomato Elongation Factor 1α (Ef1α) as reference gene for normalization (Table S1). qPCR was performed using the CFX96 Real-Time PCR machine (Bio-Rad, Hercules, CA, USA). Each 10 μL reaction contained 300 nM of each primer, 1 μL (10ng) gDNA template and 1 × iQ SYBR Green Supermix (Bio-Rad Laboratories, Hercules, CA, USA). Cycling conditions initiated with a denaturation step at 95 °C for 3 min., followed by 40 cycles of 10 sec. denaturation at 95 °C and 30 sec. annealing and extension at 60 °C, finished by a melt cycle of 0.5 °C increment per 10 sec. from 65 °C to 95 °C. Relative fold-change of the ratio between fungal and tomato gDNAs was calculated by the 2^−∆∆Ct^ method [70]. Four biological replicates and two technical replicates were used in this experiment. Tukey’s multiple comparison test was performed in order to assess significant differences between the genotypes.

### 2.3. Plant Materials 

The identified mutant M200 showing resistance to powdery mildew was either crossed with Moneymaker (MM) or backcrossed to MT to obtain F_1_ or BC_1_ seeds which were harvested from each fruit and kept separately. BC_1_ plants derived from three individual crosses (fruits) of M200 × MT were tested with powdery mildew and four plants per family were kept for self-pollination and seed production. Two of the three corresponding progenies (BC_1_S_1_) were further tested with powdery mildew and selected for seed production if showing a resistant phenotype. Three F_1_ plants from the cross M200 × MM were allowed to self-pollinate. Their progenies (F_2_) were tested with powdery mildew. The disease test and the visual inspection of further generations were performed as for the M_1_ plants. Individual F_2_ plants were selected that were MM-like in their morphology (lacking the dwarf and determinate growth characteristics of cultivar MT), and were homozygous for the *m200* allele. The selected F_2_ plants were kept for the production of F_3_ seeds and subsequently F_4_ progeny was obtained. 

**Figure 1:**
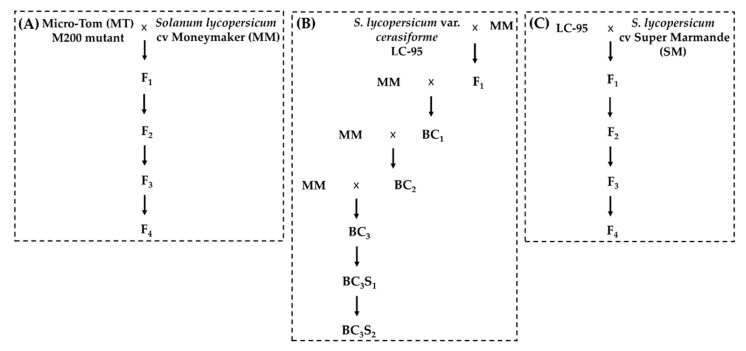
Pedigree scheme of (**A**) F_4_ plants homozygous for the *m200* allele, (**B**) BC_3_S_2_ lines homozygous for the *ol-2* allele in *Solanum lycopersicum* cv. Moneymaker background (*ol-2*_MM) and (**C**) F_4_ plants carrying the *ol-2* allele in cv. Super Marmande background (*ol-2*_SM).

For histological analysis, eight F_4_ plants carrying the *m200* allele derived from two original crosses M200 × MM were chosen (Figure 1). In addition, three plants of two BC_3_S_2_ lines derived from a cross between a resistant plant homozygous for the *ol-2* allele and MM were included (Figure 1) [48,65]. Moreover, we added three resistant F_4_ plants also carrying the *ol-2* allele derived from the self-pollination of the F_1_ from a cross between the original line LC-95 of *S. lycopersicum* var. *cerasiforme* and the cv. Super Marmande (SM) (Figure 1). For simplicity during the description of Figures and Tables, the first *ol-2* genotype is referred to as *ol-2*_MM and the second as *ol-2*_SM. Furthermore, three transgenic plants of a T_2_ family carrying the RNAi construct able to silence *SlMLO1*, *SlMLO5*, and *SlMLO8* as described in Zheng et al. [48] were selected. As susceptible control, three MM plants were included in this experiment. The transgenic plants carrying the RNAi construct were selected by standard PCR performed on DNA isolated with the 2% CTAB method [71] from all the germinated seedlings, using two primer pairs, one targeting the NPTII gene and the other the 35S promoter. Primer sequences are shown in Table S1.

### 2.4. Cloning of the SlMLO1 Coding Sequence from the Mutagenized Resistant Micro-Tom Plant M200

The third and fourth true leaves of the M200 plant and two MT plants (not subjected to the EMS treatment) were collected after the powdery mildew test and immediately frozen in liquid nitrogen. The samples were ground in liquid nitrogen with mortar and pestle. Total RNA was isolated with the RNeasy^®^ plant mini kit (Qiagen, Hilden, Germany) according to the manufacturer’s instructions. The concentration of the total RNA was measured using the Nanodrop. Approximately 1 μg of RNA was treated with DNase (Invitrogen) to remove any DNA contamination. This treated RNA was used in a one-step PCR with the SuperScript^®^ III (Invitrogen, Waltham, MA, USA) and the specific primers for the *SlMLO1* gene used in Zheng et al. [48] (sequences in Table S1). The amplified PCR products were run on a 1% agarose gel. The bands with the desired product size (1743-bp) were excised from the gel and the products recovered using the QIAquick gel extraction kit (Qiagen). The eluted PCR products were sequenced (Table S1) with primers used for amplification of the full-length cDNA as well as two primers (*SlMLO1*_seqA and *SlMLO1*_seqB) located in between and the obtained sequences aligned with the known *SlMLO1* coding sequence (cds) of Heinz (Solyc04g049090.3) using the package MegAlign of the software DNASTAR^®^ Lasergene8. The predicted protein derived from the *SlMLO1* sequence cloned from the M200 plant was analyzed using the TMHMM software (http://www.cbs.dtu.dk/services/TMHMM/, accessed on 9 November 2020) and the PROTTER web tool to predict sequence features and visualize the protein [72].

### 2.5. Development of a HRM Marker for Detection of the Mutation in the SlMLO1 Gene

In order to follow the segregation of the SNP associated with the *m200* allele in BC_1_ generation (19 plants) of the backcrosses between the M200 plant × MT, the selfing BC_1_S_1_ progenies (144 plants) and F_2_ families (115 plants) of the crosses between M200 plant × MM, the DNA of each plant was isolated using 2% CTAB in a protocol adapted for a 96-well plate [71]. The quantity and integrity of genomic DNA were determined using the Nanodrop and running 1 μL of the isolated DNA on an agarose gel (1%), respectively. 

Primers amplifying a gDNA fragment of 406-bp containing the mutation site were designed for a high-resolution melting assay (HRM). The sequences of these primers are reported in the Table S1. PCR amplifications were carried out in a 10 μL reaction mixture containing 10 ng of genomic DNA, 2 μL of 5× PCR buffer, 0.4 μL of 5 mM dNTPs, 0.5 U Phire™ Hot Start II DNA Polymerase (ThermoFisher, Waltham, MA, USA), 0.25 μM of forward and reverse primer (10 mM each) and 1 µL of LC GreenPlus (Idaho Technology Inc., Salt Lake City, UT, USA). The amplification included an initial denaturation at 98 °C for 30 s, followed by 41 cycles of 98 °C for 5 s, 60 °C for 5 s and 72 °C for 15 s, and finishing with a final elongation at 72 °C for 30 s. The HRM genotyping was performed on a Light Scanner instrument (HR96 model, Idaho technology Inc., Salt Lake City, UT, USA) with continuous melting curve acquisition (10 acquisitions per °C) during a 0.1 °C/s ramp from 40 to 95 °C. Data were retrieved and analyzed using the Light Scanner software followed by manual curation of the obtained genotype calls. DNA samples from MT or MM plants (homozygous for the wild-type *SlMLO1* allele), M200 plants (homozygous for the mutated *m200* allele) and BC_1_ (M200 × MT) or F_1_ (M200 × MM) plants (heterozygous) were used as controls to establish the reference HRM curves.

### 2.6. Histological Analysis

The powdery mildew disease assay was performed on four-week-old plants as described in Section 2.2, but using a higher concentration of *On* spores equal to 3 × 10^5^ conidia/mL. 

Samples from four plants of each genotype were collected 72 h postinoculation, bleached in a 1:3 (*v*/*v*) acetic acid/ethanol solution, stained 48 h later by boiling in 0.005% trypan blue in lactophenol: ethanol (1:2 *v*/*v*) solution for 3-5 min and finally cleared in a nearly saturated aqueous solution of chloral hydrate (5:2 *w*/*v*). Analysis was conducted using a Zeiss Axiophot bright field microscope. For quantification of fungal structures approximately 100 infection units were analyzed per genotype, from at least two different plants per genotype. An infection unit (IU) was defined as a spore with a germination tube. For each IU, the presence of haustorium or papilla was recorded. For some IU, photos were taken using the 100x magnification coupled with the differential interface contrast (DIC) technique at different focus to be able to observe all the fungal structures eventually developed.

## 3. Results

### 3.1. A Novel EMS mlo Mutant (M200) Shows Resistance to Powdery Mildew

**Figure 2:**
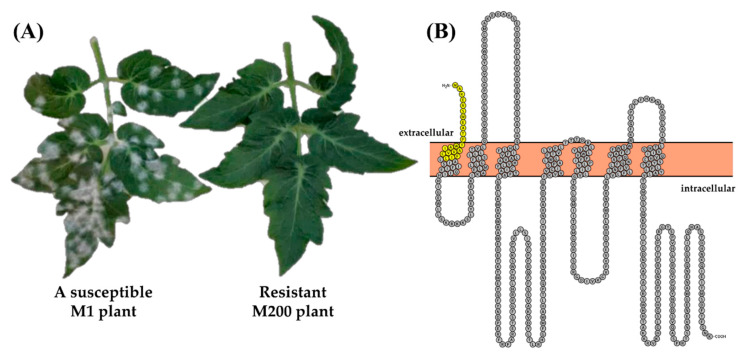
A novel EMS mlo mutant (M200) shows resistance to powdery mildew. (**A**) Contrasting phenotypes of susceptible leaves of an M_1_ plant and resistant leaves of the M200 plant after Oidium neolycopersici inoculation. (**B**) Schematic representation of the SlMLO1 protein of the cv. Heinz. The predicted truncated m200 protein is indicated in yellow, while the region that is absent in m200 is indicated in grey.

An EMS-mutagenized population of tomato cv. MT was developed and phenotypically screened for resistance to the powdery mildew pathogen *O. neolycopersici* (*On*). During the EMS treatment, the 1% *v*/*v* EMS concentration was mostly used to maximize the genomic variation with a minimum decrease in viability. The M_1_ plants derived from the first two batches of EMS treatment (about 1000 seeds per batch) were inoculated with spores of the pathogen *On* by spray inoculation. In the first group of approximately 1000 M_1_ plants, one plant (M200) showed no fungal sporulation, while all other plants were severely infected (Figure 2A). 

The M_1_ plants were allowed to self-pollinate and M_2_ seeds were collected. All the tested M200 M_2_ plants were free of PM symptoms, and thus resistant. Except for the resistant phenotype, no other morphological differences were observed in M200 M_1_ and M_2_ plants compared to wild-type MT (not subjected to the EMS treatment).

To find the causal mutation for the highly resistant phenotype of the M200 plant and its M_2_ progeny, SlMLO1 was chosen as the first candidate gene. The coding sequence (cds) of the SlMLO1 gene in M200 was obtained. A SNP (T65A; SL4.0ch04:38,795,717 coding strand position) was detected in the SlMLO1 cds of the M200 plant compared to the sequence in MT and tomato cultivar Heinz (Figure S1). This point mutation results in a premature stop codon (L22*). This stop codon at position 22 in the full-length SlMLO1 protein sequence of Heinz is located in the first transmembrane domain (Figure 2B). The resulting truncated protein contains 21 amino acids (aa) instead of 513 aa (Figure 2B). Although the N-terminal domain of the wild-type SlMLO1 protein is predicted to be extracellular, the hypothetical truncated 21 aa protein of the M200 mutant does not contain a functional transmembrane region and is predicted to be located in the intracellular space. The premature stop codon location from M200 differs from the stop codon identified in the ol-2 allele, located in the second intracellular loop [68]. This loss-of-function allele of the SlMLO1 gene is therefore novel and was named m200.

### 3.2. The m200 Allele Is a Unique SlMlo1 Loss-of-Function Allele

The T-to-A tranversion in the m200 mutant is not a typical EMS-induced mutation. In order to verify whether any natural impaired SlMLO1 allele is already present in MT, the full-length nucleotide sequence of the Heinz SlMLO1 mRNA (1878-bp) was compared with the full length transcript AK322443 (from clone LEFL1037DE09) of MT SlMLO1 (1847-bp), obtained from NCBI (https://www.ncbi.nlm.nih.gov/, accessed on 3 June 2016). Additionally, successful cloning of full-length cDNA sequence was accomplished by PCR amplification of mRNA derived from our wild-type MT plants. A multialignment of these SlMLO1 sequences did not reveal any mutation (Figure S1). Thus, these findings indicated that the SlMLO1 gene in MT does not differ from the one in other cultivated tomatoes, like Heinz and MM. In addition, we searched for any predicted mutations of the SlMLO1 gene among the sequenced 360 tomato accessions in the Tomato 360 variants SL2.50 genome browser at SGN (https://solgenomics.net/jbrowse_solgenomics/, accessed on 1 April 2021) for position SL2.50ch04:39557939 [73]. The output of this analysis also revealed that there are no predicted natural mutations at the T65 position where the m200 SNP occurs. These results suggest that the point mutation in the SlMLO1 gene in the M200 mutant is a new and unique mutation.

### 3.3. The Resistant Phenotype Fully Cosegregates with the Novel m200 Allele

To analyze the association of PM resistant phenotype with the presence of the *m200* allele, the M200 mutant was backcrossed to MT and additionally crossed to MM (Figure 1). Initially, three BC_1_ families derived from different fruits of the cross between M200 and MT were tested with *On*. All 19 BC_1_ plants (12 plants of BC_1_ family 1, three of family 2, and four of family 3) showed clear fungal sporulation, and were as susceptible as the controls, MM and MT. A high-resolution melting (HRM) marker was developed (Table S1) which could clearly distinguish the *SlMLO1* allele carried by the wild-type MT/MM from the mutated *m200* allele. All 19 BC_1_ plants were heterozygous for the *m200* allele.

Two BC_1_S_1_ families derived from M200 × MT and three F_2_ from M200 × MM were produced and their phenotypic responses to *On* were assessed (Tables S2 and S3). All BC_1_S_1_ and F_2_ resistant plants were homozygous for the *m200* allele, and all susceptible plants were either homozygous or heterozygous for the MT/MM allele. Overall, these results confirm that PM resistance cosegregates with the *m200* allele.

### 3.4. Full Resistance Provided by the m200 Allele

To compare the level of resistance conferred by the newly identified *m200* allele with other mutants of the *SlMLO1* gene, we performed a disease test where we included the *m200* mutant in MM background (F_4_ generation), the *ol-2* mutant in two different genetic backgrounds (MM and Super Marmande [SM]; Figure 1), as well as the RNAi::*SlMLO1* line in which the *SlMLO1*, *SlMLO5* and *SlMLO8* genes are silenced [48].

**Figure 3:**
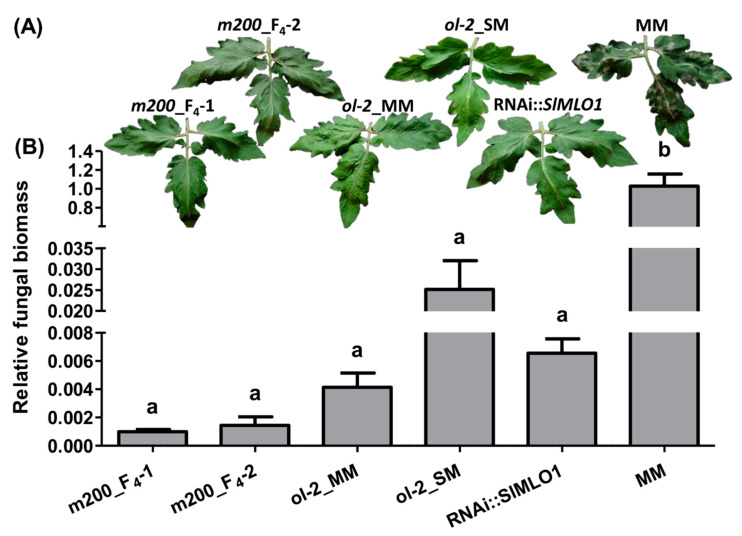
Phenotypic evaluation of the powdery mildew symptoms and relative fungal biomass quantification. Panel (**A**) shows leaves collected 18 days after the pathogen inoculation. Panel (**B**) refers to fungal biomass measured by relative quantification of the ratio between *Oidium neolycopersici* and plant gDNAs on different genotypes (F_4_ plants carrying the *m200* allele, plants carrying the *ol-2* allele in Moneymaker (MM) and Super Marmande background, a plant carrying the RNAi::*SlMLO1* construct, and MM). Bars show standard errors based on four plants. Columns labeled with different letters are significantly different at *p* < 0.05 according to Tukey’s multiple comparison test.

The control MM plants were heavily infected at 18 days post inoculation (dpi) and showed significantly higher fungal biomass when compared with all the other genotypes (Figure 3). For the *ol-2* mutant in SM background (hereafter, *ol-2*_SM), no fungal sporulation was observed on the third and fourth leaves (Figure 3A). Occasionally, weak mycelium growth could be seen on the first and second true leaves, while no fungal sporulation was observed on all plants of the *m200* mutant (hereafter *m200*_F_4_), the *ol-2* mutant in MM background (hereafter, *ol-2*_MM) and the RNAi::*SlMLO1* line, throughout the entire disease assay (Figure 3A). Compared to the other mutants, although not significant, fungal biomass was reduced in plants carrying the *m200* allele (Figure 3B). 

### 3.5. Papilla Formation Is Associated with Resistance in the m200 Mutant

A histological experiment was conducted to (1) study the resistance mechanism of the M200 resistant mutant and (2) compare the level of resistance conferred by the *m200* mutant allele in MM background with other genotypes including the *ol-2*_MM, *ol-2*_SM and RNAi::*SlMLO1* lines. 

**Figure 4:**
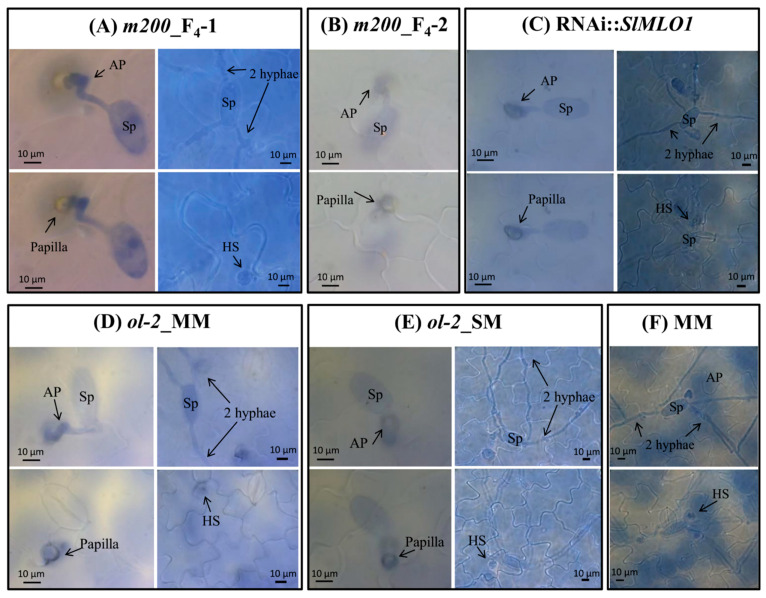
Microscopic observations on powdery mildew infection and development of the infection units (IU) of *Oidium neolycopersici* on six different genotypes. In each panel photos are taken from (**A**) and (**B**) F_4_ plants carrying the *m200* allele, (**C**) a plant carrying the RNAi::*SlMLO1* construct, (**D**) and (**E**) plants carrying the *ol-2* allele in Moneymaker (MM) and Super Marmande (SM), (**F**) MM, respectively. Photos of two IU/genotype are shown, except for the MM and F_4_-2 carrying the *m200* allele where only one IU is shown. Each photo is taken with different focus to observe all the fungal structures and papillae, from the most superficial to the deepest ones. Sp = spore, AP = appressorium; HS = haustorium; 2 hyphae = secondary hyphae.

Compared to MM, fungal growth on all individuals of the M200 mutant was considerably reduced due to the formation of a papilla beneath the appressorium (Figure 4 and Table 1). In MM, hardly any papillae were formed. In contrast, the percentage of papilla formation per infection unit (IU) was higher than 32% in the mutant genotypes, varying from 32.7% in the *m200*_F_4_-2 to 71.6% in RNAi::*SlMLO1* (Figure 4 and Table 1). Simultaneously, the percentage of haustorium formation per infection unit was drastically decreased in all the tested mutant genotypes compared to MM. No haustoria were observed in *m200*_F_4_-2, while in the other mutants haustoria were observed at a rate of 10.3% in *m200*_F_4_-1, 4.4% in *ol-2*_MM, 11% in *ol-2*_SM, and 5.5% in RNAi::*SlMLO1* (Figure 4 and Table 1). 

## 4. Discussion

Powdery mildew disease can be a problem in greenhouses and field tomato cultivations. The humidity that forms at the leaf surface when cold nights change to warm days or when plants are grown in crowded locations without sufficient air circulation is enough to ignite an infection [74]. The availability of resistant cultivars is, therefore, essential to control this disease in a sustainable way. The resistance can be achieved in several ways. Although for crop improvement mainly conventional breeding methods are used, major limitations such as lack of genetic diversity are frequently observed in the domesticated tomato [9]. Thus, genetic modification technologies including genome editing approaches are considered as an extension of traditional breeding methods. However, the deployment of transgenic plants in plant breeding and agriculture is still socially and politically debated in many parts of the world. In Europe, the plants obtained with genome editing tools are subjected to the same stringent regulations as transgenic organisms [75]. Therefore, currently, nontransgenic strategies are favored to uncover novel alleles. One of the ways consists of inducing mutations in PM susceptibility genes artificially with chemical mutagens, such as EMS. 

In this work, we describe the set-up of an EMS mutant population of the tomato cv. MT with which we aimed at finding new sources of resistance to various diseases. Here we focused in particular on finding sources of resistance to the PM disease caused by *O. neolycoperisici*. By screening the EMS plants, a new loss-of-function allele of the *SlMLO1* gene, designated *m200* was identified, which confers full resistance against PM. Histological study showed that the resistance of the M200 mutant is associated with papilla formation. 

### 4.1. Is the m200 Mutation a Real Product of the EMS Mutagenesis?

A PM disease test was performed on the M_1_ plants initially obtained with the intention of finding dominant mutations. The M200 mutant was found, and the sequence analysis showed that the resistance was due to a nonsense mutation (T65A) leading to a stop codon in the coding region of the *SlMLO1* gene (Figure 2B). It is unexpected that a recessive mutation occurred in homozygous state in an M_1_ plant since the probability of having a mutation on both alleles has been shown to be extremely low [76]. The *m200* allele seems to be a real product of the EMS treatment given that the *SlMLO1* gene in wild-type MT is identical with the one in cultivated tomatoes, Heinz and MM. This is to be expected since MT originated from two cultivated tomatoes [20]. 

However, it is important to notice that the mutation detected in the *m200* allele is not typically produced by the EMS mutagen. EMS treatment mainly triggers transitions, e.g., purine replaced by purine A ↔ G, and pyrimidine replaced by pyrimidine C ↔ T (indicated as G/C → A/T, [77,78]). In *Arabidopsis*, almost all the EMS mutations described correspond to G/C to A/T transitions [12]. So far, EMS *mlo* null alleles were reported in wheat (16 *mlo* alleles), barley (11), and petunia (2) [60,61,64]. In all cases, except three, the mutagenized treatment produced the expected base substitutions (G/C → A/T). In contrast, the barley mutants *mlo-13* (T → A) [79], *mlo-26* (T → A) [79] and *mlo-30* (A → T) [36] are characterized by transversions (purine replaced by a pyrimidine, and vice-versa), as observed in *m200* (T → A). In *mlo-13* and *mlo-2*6, the transversion caused two missense mutations, V30E and L27H respectively, which in both cases lead to the loss-of-function of the protein. In *mlo-30*, the mutation occurred in intron sequences which affected transcript splicing [36] and resulted in one transcript containing an 18-nucleotide deletion of exon 12 and another containing the entire unspliced intron 11. Therefore, although not common, the type of mutation observed in the M200 mutant is not an exception. 

The fact that the mutation occurred homozygously in an M_1_ plant, can also lead to the hypothesis that it spontaneously occurred. Spontaneous mutations in *Arabidopsis* are known to take place at a rate of 10^−7^ to 10^−8^ bp/generation [80,81]. However, the large majority of spontaneous mutations are transition mutations [82]. The occurrence of the *m200* mutation might also be explained by a gene conversion event involving a paralog *SlMlo* gene or a point mutation created at the break point of a gene conversion event [83]. 

### 4.2. Is the Resistance Level of Slmlo Mutants Dependent on Papilla Formation?

The *Slmlo1* mutants and silenced plants showed a large increase in the percentage of IU to which the plant had responded with the formation of a papilla, compared to the susceptible control MM plants (Table 1). This is in line with observations in other *mlo* mutants [26,69,84]. Consonni et al. [46] reported that the *mlo*-based resistance is characterized at the cellular level by the timely cell-wall deposition of papillae at the attempted fungal penetration sites which lead to early termination of fungal infection. However, more recent reports on *mlo* mutants or silenced plants show that papillae can be observed in both resistant and susceptible plants, and a distinction should be made between effective and ineffective papillae [85]. In apple, papillae are larger in resistant *mlo* lines than in susceptible wild-type lines [55]. In barley, effective papillae show higher concentrations of callose, arabinoxylan and cellulose than ineffective papillae [85]. Nevertheless, callose deposition in papillae is not required for *mlo*-mediated penetration resistance [86]. In *Arabidopsis*, PMR4/GSL5 callose synthase (POWDERY MILDEW RESISTANCE4/GLUCAN SYNTHASE-LIKE5) is responsible for the spontaneous callose deposition in *mlo2* mutant, however, no differences in the level of PM resistance were observed between *mlo2* single and *mlo2 pmr4* double mutants [86]. In addition, it was shown that resistance conferred by *mlo* is not dependent on salicylic acid (SA) accumulation [86]. Further research is required to assess the importance of papillae formation for *mlo*-based resistance.

### 4.3. Is the Level of mlo-Based Resistance Influenced by the Position of the Mutation? 

The full resistance of the *m200* plant is hypothesized to be caused by the severe truncation of this mutant *Slmlo1* allele. After reviewing the available literature on barley *mlo* mutants, three interesting cases, namely *mlo-13*, *mlo-17* and *mlo-32*, were found [79,87,88]. All three mutants carry mutations leading to a stop codon in the first transmembrane of the HvMlo protein, which corresponds to the same region where the *m200* mutation is found. They were all indicated as completely resistant mutants. Moreover, another barley mutant, the *mlo-43*, was found to carry a stop codon in the second intracellular domain, the same as the nonsense mutation identified in tomato *ol-2* mutant [60]. The *mlo-43* is a mutant of the cv. Bonus and it was also described as completely resistant [89]. A mutant of the same cultivar, *mlo-36*, was described to contain a nonsense mutation at W357, in the sixth transmembrane domain [60,89]. Both mutants were only phenotypically scored, and considered highly resistant, with *mlo-36* even annotated as immune [89]. 

Other more recent evaluations of barley impaired alleles have not been found due to premature protein truncation. The reason is that it was shown that defective protein variants would probably not pass the quality test of the ERAD machinery (endoplasmic reticulum-associated protein degradation, [90]). The ER-localized quality control system monitors and validates proper folding and modification of proteins, among which the membrane proteins. If this holds true, the extremely truncated m200 protein, as well as the ol-2 variant, should be subjected to a dramatic reduction in accumulation. Thus, both variants should lead to a similar level of resistance, if compared in the same background. Though it is currently largely unknown which signatures classify malformed membrane proteins, previous studies indicated that the second cytoplasmic loop and the transmembrane regions are the major quality determinant of the HvMlo protein variants [60,90]. Premature truncations heavily affect protein folding. Therefore, mutants containing amino acid substitutions were preferred to truncated *mlo* alleles in studies addressed at evaluating the biological activity of the *Mlo* variant.

**Figure 5:**
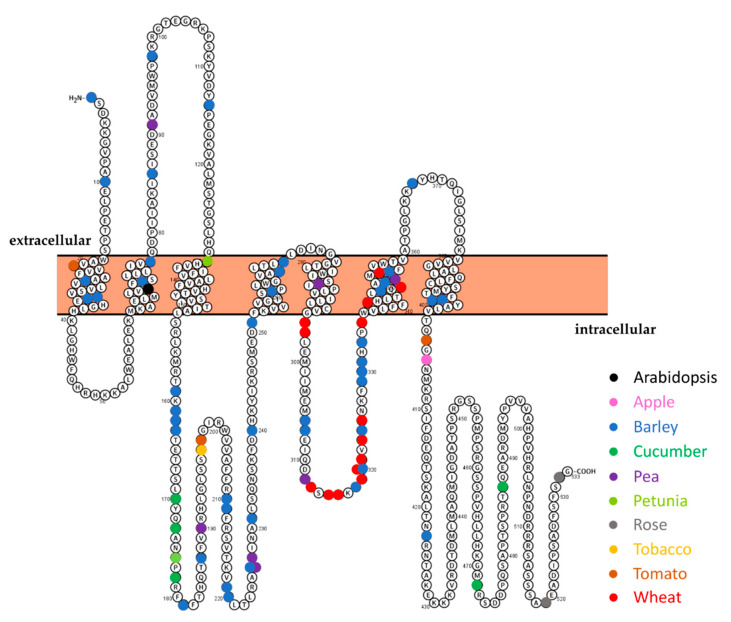
Schematic representation of the complete barley HvMLO protein. The orange bar represents the plant membrane. Colored dots indicate the amino acids of the corresponding *mlo*-mutants in different plant species. Overview of the depicted *mlo*-mutants are shown in Table S4.

Alternatively, it is possible to exploit amino acid residues that are crucial for the powdery mildew susceptibility-conferring function of the MLO protein (Figure 5, Figure S2). With the increasing number of *MLO* sequences being functionally characterized in various plant species, multiple protein alignments point out the occurrence of highly conserved residues/regions (Figure S2). These amino acids have been previously shown to be invariable in MLO orthologs involved in the interaction with PM fungi and therefore predicted to have an important functional/structural role for the PM susceptibility-conferring function [42,91]. In addition, a codon-based evolutionary analysis was conducted that resulted in the identification of 130 codons under negative selection, thus predicted to be conserved during evolution (Figure S2) [92]. Amino acids specific for monocot and dicot MLO proteins which do not seem to influence the interaction with PM pathogens were also highlighted (Figure S2). Mining the available literature revealed 21 naturally occurring *mlo* alleles as well as 74 chemically and radiation-induced *mlo* alleles, comprising 77 single amino acid substitutions that result in loss-of-*MLO* function (Table S4). We combined the information of amino acids with the actual mutations found in natural and artificial mutants to map functionally important sites of the MLO protein as being sensitive to functional impairment by mutational perturbation (Figure 5, Table S4). The large majority of the mutations are found in the second (21) and third (23) cytoplasmic domains, which have already been identified as relevant regions for the MLO proteins acting as PM-susceptibility factors (Figure 5). Transmembrane (TM) regions are additional sites of loss-of-function mutations in 24 cases, with the predominant occurrence in the sixth transmembrane (7) (Figure 5), indicating that TM domains harbor important sites for protein conformational changes. These sites/regions are critical for the susceptibility-conferring activity of the MLO protein. 

Any novel MLO protein characterized in a certain crop species can be added to this alignment provided by Figure S2 to select predicted amino acid positions that, being under negative selection, can represent targets of protein loss of function. If artificial or natural mutants are not available, the information of Figure 5 can be usefully coupled with the genome editing technologies to obtain loss-of-function mutations, especially within the protein domains/sites that act as determinants of PM susceptibility.

## 5. Conclusions

The use of impaired *MLO* genes in plant breeding against PM is a promising strategy due to its broad-spectrum and durable characteristics. In this study, we developed a new genetic resource in MT background by means of EMS mutagenesis, and transferred it to a genetic background resembling MM by means of crossing and selfing. The mutant M200 containing a novel allele (the *m200* allele) of the tomato *SlMLO1* gene showed profound PM resistance with no fungal sporulation and hardly detectable fungal biomass. Thus, it represents a valuable new mutant allele that can be used in breeding PM-resistant tomato cultivars. 

## Figures and Tables

**Table 1:** *Oidium neolycopersici* development 72 h after the artificial inoculation. Approximately 100 infection units (IU = fungal spore producing a germination tube) per genotype were observed and the number of papillae and haustoria were counted. Subsequently, the percentage of IU showing a papilla or haustorium was calculated.

Genotype	Number of Fungal/Plant Structures Observed			%Papilla/IU	%Haustorium/IU
	IU	Papilla	Haustorium		
*m200*_F_4_-1	97	34	10	35.1	10.3
*m200*_F_4_-2	101	33	0	32.7	0
*ol-2*_MM	90	55	4	61.1	4.4
*ol-2*_SM	100	51	11	51	11
RNAi::*SlMLO1*	109	78	6	71.6	5.5
MM	102	1	92	0.98	90.2

## Data Availability

The data presented in this study are available in the article and supplementary material.

## Supplementary Materials

The following are available online at https://www.mdpi.com/article/10.3390/genes12050719/s1, Figure S1: Nucleotide alignment of the *SlMLO1* sequence experimentally obtained from the tomato cv. Micro-Tom (MT), the full-length transcript AK322443 of MT *SlMLO1* obtained from NCBI, the *SlMLO1* gene in M200, MM leaf cDNA sequence of *SlMLO1* obtained from Zheng et al. [22] and the one from the cv. Heinz as in the SGN database (Solyc04g49090.3). Arrow indicates the base change T → A responsible for the premature stop codon in M200 plant, Figure S2: Protein alignment of functionally characterized MLO sequences of *Arabidopsis* thaliana AtMLO2, -6, and -12 (GenBank accession numbers NP172598, NP176350, and NP565902) [17], *Pisum sativum* (pea) PsMLO1 (GenBank accession number FJ463618) [1], *Medicago truncatula* (barrel clover) MtMLO1 (GenBank accession number HQ446457) [1], *Lotus japonicus* LjMLO1 (GenBank accession number AY967410) [1], *Capsicum annuum* (pepper) CaMLO2 (GenBank accession number AFH68055) [23], *Cucumis sativus* (cucumber) CsaMLO1 and -8 (GenBank accession numbers Csa1M085890.1 and Csa5M623470.1) [12,14], *Solanum lycopersicum* (tomato) SlMLO1 (GenBank accession number NP001234814) [7], *Nicotiana tabacum* (tobacco) NtMLO1 (GenBank accession number KM244716) [15], *S. melongena* (eggplant) SmMLO1 (GenBank accession number KM244717) [24], *Malus domestica* (apple) MdMLO19 (GenBank accession number MDP0000168714) [10], *Triticum aestivum* (wheat) TaMLO-A1b, TaMLO-B1a, and TaMLO-D1 (GenBank accession numbers AX063298, AF361932, and AX063296) [25,26], *Hordeum vulgare* (barley) HvMLO (GenBank accession number Z83834) [27] and *Oryza sativa* (rice) OsMLO2 (GenBank accession number AF384030) [25]. Highlighted in green and in light blue are the conserved amino acids among the whole MLO family indicated by Kusch et al. [28] and by Elliott et al. [29], respectively. Amino acids highlighted in gray refer to the ones reported to be under negative selection by Appiano et al. [30]. Letters displayed in green, light blue or gray indicate synonymous amino acid exchanges in each of three categories above described. Letters in red bold indicate amino acids identified in *mlo*-mutants for each of the plant species described above. Black lines indicate the position of the transmembrane domains which have been numbered with romans numbers, Table S1: Primer pairs used in this study, Table S2: Genotyping and phenotyping of eight progenies (BC_1_S_1_) derived from two (i.e., BC_1__1 and BC_1__3) of the three BC_1_ crosses M200 × MT, Table S3: Genotyping and phenotyping of the progenies (F_2_) of three crosses between the resistant M200 plant and the tomato cv. Moneymaker (M200 × MM), Table S4: Overview of the *mlo*-mutants described in the literature.
