# Supplementary material for: Discovery and Characterization of a Novel Tomato mlo Mutant from an EMS Mutagenized Micro-Tom Population
Source: Genes (Basel). 2021 May 11;12(5):719. doi: 10.3390/genes12050719 (PMC8150974; doi:10.3390/genes12050719)
Supplement: Supplementary file 1 [file genes-12-00719-s001.zip › genes-1198661-supplementary.pdf]

genes

MDPI

Article

Discovery and Characterization of a Novel Tomato *mlo* Mutant from an EMS Mutagenized Micro-Tom Population

Zhe Yan †, Michela Appiano †, Ageeth van Tuinen, Fien Meijer-Dekens, Danny Schipper, Dongli Gao, Robin Huibers, Richard G. F. Visser, Yuling Bai and Anne-Marie A. Wolters *

Supplementary Materials

Table S1. Primer pairs used in this study.

Primer name	Forward primer (5′- 3′)	Reverse primer (5′- 3′)	PCR product size
*SlMLO1*_full-length	TTGACATTTCCCCTTCTTCTTA	TACAAAATCATTGCCATTTGAA	1743 bp
*SlMLO1*_seqA	CCCTTTCTGAAATCCTTACTCC		
*SlMLO1*_seqB	TATTGCTAGTTGGCACAAAACT		
HRM_marker	TGGCTAAAGCACGGTCTA	CTGGATCTTGCAACACTGTCA	406 bp
35S_promoter	GCTCCTACAAATGCCATCA	GATAGTGGGATTGTGCGTCA	195 bp
NPTII_marker	TCGGCTATGACTGGGCACAAC	AAGAAGGCGATAGAAGGCGA	722 bp
SlEf1α	ACAGGCGTTCAGGTAAGGAA	GAGGGTATTCAGCAAAGGTCTC	120 bp
On_ITS	CGCCAAAGACCTAACCAAAA	AGCCAAGAGATCCGTTGTTG	90 bp

Table S2. Genotyping and phenotyping of eight progenies (BC_1S_1) derived from two (i.e. BC_1_1 and BC_1_3) of the three BC_1 crosses M200 × MT.

M200 x MT	Number of susceptible plants	Number of resistant plants	Expected 3S:1R		HRM profiles		
			X^2	*p*	Wild-type allele	heterozygous	*m200* allele
BC_1S_1_1-1	10	6	2.07	0.15	2	8	6
BC_1S_1_1-2	13	4			4	9	4
BC_1S_1_1-4	13	6			3	10	6
BC_1S_1_1-10	12*	7			4	7	7
BC_1S_1_3-1	14	4	0.45	0.50	4	10	4
BC_1S_1_3-6	11	7			4	7	7
BC_1S_1_3-7	17	2			4	13	2
BC_1S_1_3-9	11	8			2	9	8

S = susceptible, R = resistant.
* One plant could not be genotyped with the HRM marker.

Table S3. Genotyping and phenotyping of the progenies (F_2) of three crosses between the resistant M200 plant and the tomato cv Moneymaker (M200 × MM).

M200 x MM	Number of susceptible plants	Number of resistant plants	Expected 3S:1R		HRM marker profiles		
			X2	p	Wild-type allele	heterozygous	*m200* allele
F_2_1	25	14	2.47	0.12	9	16	14
F_2_2	27	11	0.32	0.57	11	16	11
F_2_3	25*	14	2.47	0.12	5	19	14

S = susceptible, R = resistant.
* One plant could not be genotyped with the HRM marker.

Table S4. Overview of the *mlo*-mutants described in the literature.

Plant species	*MLO* gene	Allele name	Amino-acid exchange	Location	Origin	Reference
Pea (*Pisum sativum*)	*PsMLO1*	*er1-1*	S227 → stop	2IC	natural origin	[1]
		er1-2	aberrant splicing variants	n.a.	natural origin	
		er1-3	G288 → stop	TM4	natural origin	
		er1-4	ΔA91 (in frame shift)	miss 1EXTRA &TM1	natural origin	
		er1-5	W190 → stop	2IC	DES	[2]
		er1-6	L353P	TM6	natural origin	[3]
		n.a.	Q226 → stop	2IC	ENU	[4]
		n.a.	312Y → stop	TM5	ENU	
Petunia (*Petunia* x *hybrida*)	*PhMLO1*	n.a.	S130L	TM3	EMS/TILLING	[5]
		n.a.	G176E	2IC	EMS/TILLING	
Rose (*Rosa multiflora*)	*RhMLO4*	n.a.	V519 → stop	C-terminal	natural origin	[6]
		n.a.	T532 → stop	C-terminal	natural origin	
Tomato (*Solanum lycopersicum*)	*SlMLO1*	*m200*	L22 → stop	TM1	EMS	Present study
		ol-2	Q198 → stop	2IC	natural origin	[7]
		Slmlo1.1	M403 → stop	3EXTRA	natural origin	[8]
		n.a.	Small deletion	TM7	CRISPR-Cas9	[9]
Apple (*Malus domestica*)	*MdMLO19*	n.a.	E405 → stop	3EXTRA	natural origin	[10]
Grapevine (*Vitis vinifera*)	*VvMLO3*	n.a.	INDEL	3EXTRA	CRISPR-Cas9	[11]
Cucumber (*Cucumis sativus*)	*CsaMLO1*	n.a.	V170G	TM3	natural origin	[12]
			V472I	C-terminal	natural origin	
			V557I	C-terminal	natural origin	
	CsaMLO8	n.a.	K178 → stop	2IC	natural origin	[13]
			K487 → stop	C-terminal	natural origin	
			Δ174	3IC, part of TM5 & TM6	natural origin	[14]
Tobacco (*Nicotiana tabacum*)	*NtMLO1*	n.a.	Q198R	2IC	PCR error during cloning	[15]
		n.a.	6 aberrant splicing variants	2IC	natural origin	[16]
	NtMLO2	n.a.	2 aberrant splicing variants		natural origin	
Arabidopsis thaliana	*AtMLO2*	*Atmlo2-8* (*pmr2-2*)	G66R	TM2	EMS	[17]
Barley (*Hordeum vulgare*)	*HvMLO*	n.a.	V76L	TM2	natural origin	[18]
		mlo-12	F240L	2IC	NMU	
		mlo-28	T222I	2IC	NaN3	
		mlo-1	W162R	2IC	X-rays	
		mlo-6	3 aberrant splicing variants	n.a.	EMS	
		mlo-44	1 aberrant splicing variant	n.a.	NaN3	
		mlo-16	1 aberrant splicing variant	n.a.	EMS	
		mlo-30	2 aberrant splicing variants	C terminal	EMS	
		mlo-11	aberrant splicing variants	n.a.	natural origin	
		mlo-2	A349T	TM6	X-rays	
		mlo-34	W423 → stop	C-terminal, CaMBD	EHOES	
		mlo-36	W357 → stop	TM6	iso-PMS	
		mlo-39	Q351 → stop	TM6	iso-PMS	
		mlo-43	Q210 → stop	2IC	NaN3	
		mlo-32	E35 → stop	TM1	NaN3	
		mlo-31	G276 → stop	TM5	NaN3	
		mlo-35	H231L	2IC	iso-PMS	
		mlo-37	S71F	1EXTRA	iso-PMS	
		mlo-38	G318R	3IC	iso-PMS/NaN3	
		mlo-40	G264D	TM4	NaN3	
		mlo-41	R209K	2IC	NaN3	
		mlo-42	S187L	2IC	X-rays	
		mlo-5	M1I	N-terminus	EMS	
		mlo-8	M1V	N-terminus	EMS	
		mlo-7	G226D	2IC	EMS	
		mlo-9	R10W	N-terminus	EMS	
		mlo-13	V30E	TM1	EMS	

Plant species	*MLO* gene	Allele name	Amino-acid exchange	Location	Origin	Reference
Barley (*Hordeum vulgare*)	*HvMLO*	*mlo-17*	S31F	TM1	EMS	[18]
		mlo-26	L270H	TM4	EMS	
		mlo-27	G318E	3IC	EMS	
		mlo-29	P334L	3IC	NaN3	
		mlo-33	A306T	3IC	NaN3	
		mlo-3	frame shift P396	TM7	gamma-rays	
		mlo-4	frame shift W159	2IC	X-rays	
		mlo-10	deletion F182 and T183	2IC	gamma-rays	
		n.a.	W159R	2IC	SDM	
		n.a.	W162A or W163E	2IC	SDM	
		n.a.	E163R	2IC	SDM	
		n.a.	S187F	2IC	SDM	
		n.a.	D219	2IC	SDM	
		n.a.	D251N	TM4	SDM	
		n.a.	L307A	3IC	SDM	
		n.a.	P320A	3IC	SDM	
		n.a.	P324A	3IC	SDM	
		n.a.	F329A	3IC	SDM	
		n.a.	W330A	3IC	SDM	
		n.a.	F331A	3IC	SDM	
		n.a.	R333A	3IC	SDM	
		n.a.	C367A	3EXTRA	SDM	
		n.a.	C98A	1EXTRA	SDM	
		n.a.	C86A	1EXTRA	SDM	
		n.a.	C114A	1EXTRA	SDM	
		n.a.	P395G	TM7	SDM	
		mlo-11(*cnv2*)	aberrant splicing variants	n.a.	natural origin	[19]
Wheat (*Triticum aestivum*)	*TaMLO-A1*		P325L	3IC	EMS/TILLING	[20]
	TaMLO-A1		A354V	TM6	EMS/TILLING	
	TaMLO-B1		G296E	TM5	EMS/TILLING	
	TaMLO-B1		T297I	3IC	EMS/TILLING	
	TaMLO-B1		R313W	3IC	EMS/TILLING	
	TaMLO-B1		S315N	3IC	EMS/TILLING	
	TaMLO-B1		G319R	3IC	EMS/TILLING	
	TaMLO-B1		A320T	3IC	EMS/TILLING	
	TaMLO-B1		T345M	TM6	EMS/TILLING	
	TaMLO-D1		V316T	3IC	EMS/TILLING	
	TaMLO-D1		G319R	3IC	EMS/TILLING	
	TaMLO-D1		A320T	3IC	EMS/TILLING	
	TaMLO-D1		P321S	3IC	EMS/TILLING	
	TaMLO-D1		V323I	3IC	EMS/TILLING	
	TaMLO-D1		P335L	3IC	EMS/TILLING	
	TaMLO-D1		T345M	TM6	EMS/TILLING	
	TaMLO-A1		aberrant splicing variants and T78K	2IC	CRISPR-Cas9	[21]

n.a., not available; TM: Transmembrane; IC: Intracellular; EXTRA: Extracellular; DES: diethyl sulfate; ENU: N-ethyl-N-nitrosourea; EMS: Ethyl methanesulfonate; NMU: N-Nitroso-N-methylurea; NaN3: sodium azide; SDM: site-directed mutagenesis; EHOES: Ethyl hydroxyethanesulfonate; iso-PMS: isopropyl-methane-sulfonate; INDEL: insertion and deletion.

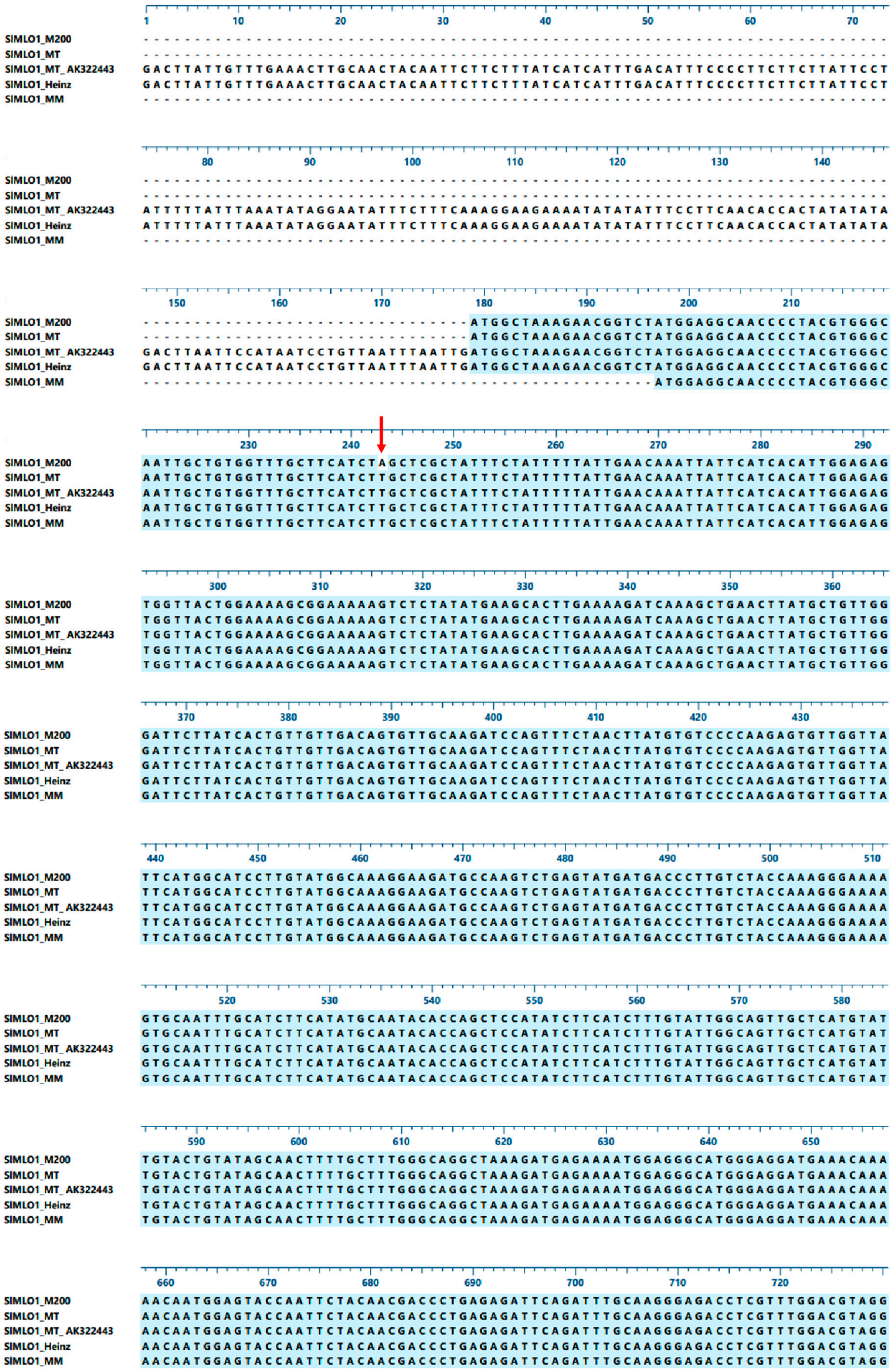
SlMLO1_M200
SlMLO1_MT
SlMLO1_MT_AK322443
SlMLO1_Heinz
SlMLO1_MM

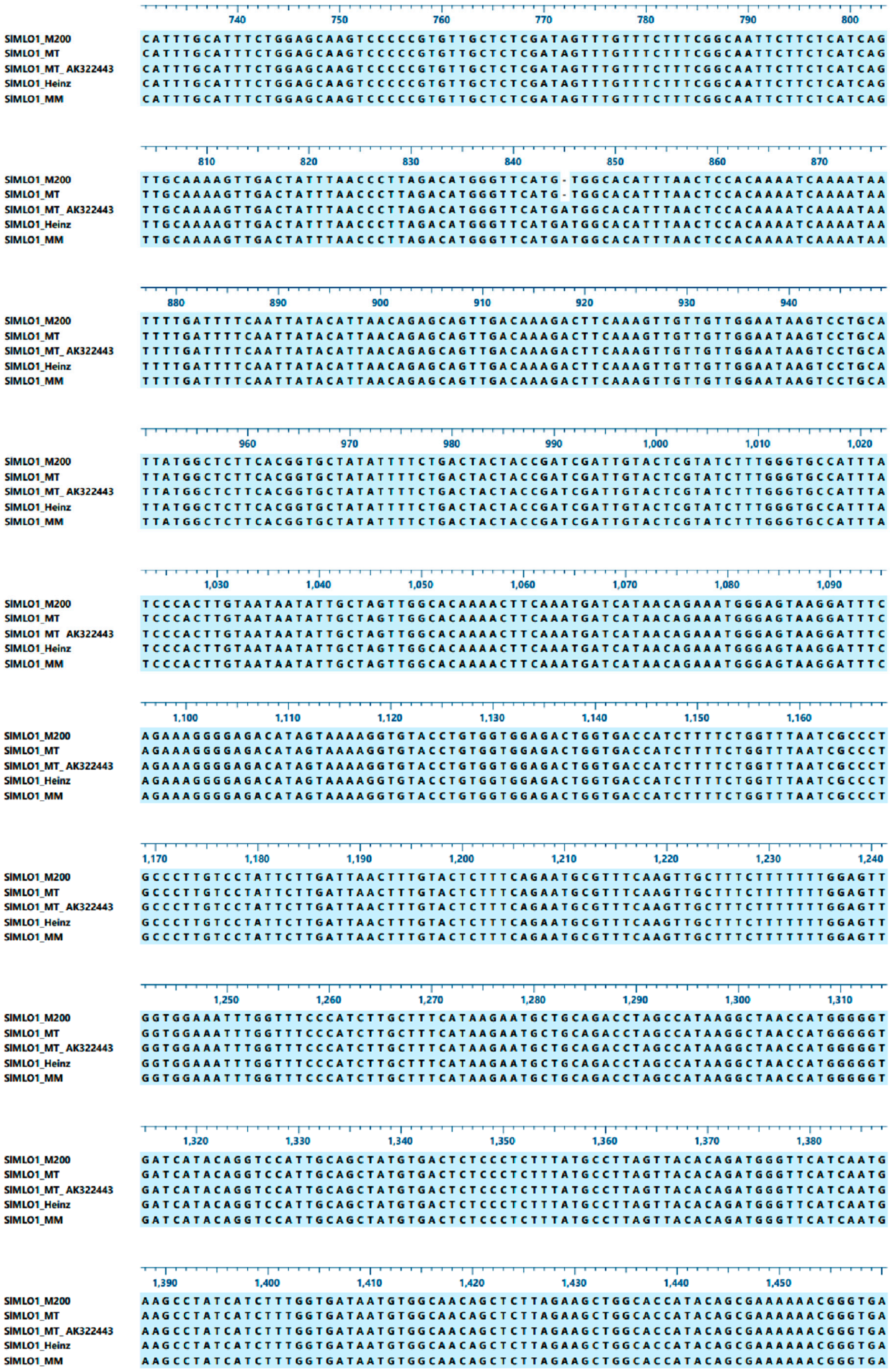
740 750 760 770 780 790 800
SlMLO1_M200 CATTTGCATTTCTGGAGCAAGTCCCCCGTGTTGCTCTCGATAGTTTGTTTCTTTCGGCAATTCTTCTCATCAG
SlMLO1_MT CATTTGCATTTCTGGAGCAAGTCCCCCGTGTTGCTCTCGATAGTTTGTTTCTTTCGGCAATTCTTCTCATCAG
SlMLO1_MT_ AK322443 CATTTGCATTTCTGGAGCAAGTCCCCCGTGTTGCTCTCGATAGTTTGTTTCTTTCGGCAATTCTTCTCATCAG
SlMLO1_Heinz CATTTGCATTTCTGGAGCAAGTCCCCCGTGTTGCTCTCGATAGTTTGTTTCTTTCGGCAATTCTTCTCATCAG
SlMLO1_MM CATTTGCATTTCTGGAGCAAGTCCCCCGTGTTGCTCTCGATAGTTTGTTTCTTTCGGCAATTCTTCTCATCAG
810 820 830 840 850 860 870
SlMLO1_M200 TTGCAAAAGTTGACTATTTAACCCTTAGACATGGGTTCATG-TGGCACATTTAACTCCACAAAATCAAAATAA
SlMLO1_MT TTGCAAAAGTTGACTATTTAACCCTTAGACATGGGTTCATG-TGGCACATTTAACTCCACAAAATCAAAATAA
SlMLO1_MT_ AK322443 TTGCAAAAGTTGACTATTTAACCCTTAGACATGGGTTCATGATGGCACATTTAACTCCACAAAATCAAAATAA
SlMLO1_Heinz TTGCAAAAGTTGACTATTTAACCCTTAGACATGGGTTCATGATGGCACATTTAACTCCACAAAATCAAAATAA
SlMLO1_MM TTGCAAAAGTTGACTATTTAACCCTTAGACATGGGTTCATGATGGCACATTTAACTCCACAAAATCAAAATAA
880 890 900 910 920 930 940
SlMLO1_M200 TTTTGATTTTCAATTATACATTAACAGAGCAGTTGACAAAGACTTCAAAGTTGTTGTTGGAATAAGTCCTGCA
SlMLO1_MT TTTTGATTTTCAATTATACATTAACAGAGCAGTTGACAAAGACTTCAAAGTTGTTGTTGGAATAAGTCCTGCA
SlMLO1_MT_ AK322443 TTTTGATTTTCAATTATACATTAACAGAGCAGTTGACAAAGACTTCAAAGTTGTTGTTGGAATAAGTCCTGCA
SlMLO1_Heinz TTTTGATTTTCAATTATACATTAACAGAGCAGTTGACAAAGACTTCAAAGTTGTTGTTGGAATAAGTCCTGCA
SlMLO1_MM TTTTGATTTTCAATTATACATTAACAGAGCAGTTGACAAAGACTTCAAAGTTGTTGTTGGAATAAGTCCTGCA
960 970 980 990 1,000 1,010 1,020
SlMLO1_M200 TTATGGCTCTTCACGGTGCTATATTTTCTGACTACTACCGATCGATTGTACTCGTATCTTTGGGTGCCATTTA
SlMLO1_MT TTATGGCTCTTCACGGTGCTATATTTTCTGACTACTACCGATCGATTGTACTCGTATCTTTGGGTGCCATTTA
SlMLO1_MT_ AK322443 TTATGGCTCTTCACGGTGCTATATTTTCTGACTACTACCGATCGATTGTACTCGTATCTTTGGGTGCCATTTA
SlMLO1_Heinz TTATGGCTCTTCACGGTGCTATATTTTCTGACTACTACCGATCGATTGTACTCGTATCTTTGGGTGCCATTTA
SlMLO1_MM TTATGGCTCTTCACGGTGCTATATTTTCTGACTACTACCGATCGATTGTACTCGTATCTTTGGGTGCCATTTA
1,030 1,040 1,050 1,060 1,070 1,080 1,090
SlMLO1_M200 TCCCACTTGTAATAATATTGCTAGTTGGCACAAAACTTCAAATGATCATAACAGAAATGGGAGTAAGGATTTC
SlMLO1_MT TCCCACTTGTAATAATATTGCTAGTTGGCACAAAACTTCAAATGATCATAACAGAAATGGGAGTAAGGATTTC
SlMLO1 MT AK322443 TCCCACTTGTAATAATATTGCTAGTTGGCACAAAACTTCAAATGATCATAACAGAAATGGGAGTAAGGATTTC
SlMLO1_Heinz TCCCACTTGTAATAATATTGCTAGTTGGCACAAAACTTCAAATGATCATAACAGAAATGGGAGTAAGGATTTC
SlMLO1_MM TCCCACTTGTAATAATATTGCTAGTTGGCACAAAACTTCAAATGATCATAACAGAAATGGGAGTAAGGATTTC
1,100 1,110 1,120 1,130 1,140 1,150 1,160
SlMLO1_M200 AGAAAGGGGAGACATAGTAAAAGGTGTACCTGTGGTGGAGACTGGTGACCATCTTTTCTGGTTTAATCGCCCT
SlMLO1_MT AGAAAGGGGAGACATAGTAAAAGGTGTACCTGTGGTGGAGACTGGTGACCATCTTTTCTGGTTTAATCGCCCT
SlMLO1_MT_ AK322443 AGAAAGGGGAGACATAGTAAAAGGTGTACCTGTGGTGGAGACTGGTGACCATCTTTTCTGGTTTAATCGCCCT
SlMLO1_Heinz AGAAAGGGGAGACATAGTAAAAGGTGTACCTGTGGTGGAGACTGGTGACCATCTTTTCTGGTTTAATCGCCCT
SlMLO1_MM AGAAAGGGGAGACATAGTAAAAGGTGTACCTGTGGTGGAGACTGGTGACCATCTTTTCTGGTTTAATCGCCCT
1,170 1,180 1,190 1,200 1,210 1,220 1,230 1,240
SlMLO1_M200 GCCCTTGTCCTATTCTTGATTAACTTTGTACTCTTTCAGAATGCGTTTCAAGTTGCTTTCTTTTTTTGGAGTT
SlMLO1_MT GCCCTTGTCCTATTCTTGATTAACTTTGTACTCTTTCAGAATGCGTTTCAAGTTGCTTTCTTTTTTTGGAGTT
SlMLO1_MT_ AK322443 GCCCTTGTCCTATTCTTGATTAACTTTGTACTCTTTCAGAATGCGTTTCAAGTTGCTTTCTTTTTTTGGAGTT
SlMLO1_Heinz GCCCTTGTCCTATTCTTGATTAACTTTGTACTCTTTCAGAATGCGTTTCAAGTTGCTTTCTTTTTTTGGAGTT
SlMLO1_MM GCCCTTGTCCTATTCTTGATTAACTTTGTACTCTTTCAGAATGCGTTTCAAGTTGCTTTCTTTTTTTGGAGTT
1,250 1,260 1,270 1,280 1,290 1,300 1,310
SlMLO1_M200 GGTGGAAATTTGGTTTCCCATCTTGCTTTCATAAGAATGCTGCAGACCTAGCCATAAGGCTAACCATGGGGGT
SlMLO1_MT GGTGGAAATTTGGTTTCCCATCTTGCTTTCATAAGAATGCTGCAGACCTAGCCATAAGGCTAACCATGGGGGT
SlMLO1_MT_ AK322443 GGTGGAAATTTGGTTTCCCATCTTGCTTTCATAAGAATGCTGCAGACCTAGCCATAAGGCTAACCATGGGGGT
SlMLO1_Heinz GGTGGAAATTTGGTTTCCCATCTTGCTTTCATAAGAATGCTGCAGACCTAGCCATAAGGCTAACCATGGGGGT
SlMLO1_MM GGTGGAAATTTGGTTTCCCATCTTGCTTTCATAAGAATGCTGCAGACCTAGCCATAAGGCTAACCATGGGGGT
1,320 1,330 1,340 1,350 1,360 1,370 1,380
SlMLO1_M200 GATCATACAGGTCCATTGCAGCTATGTGACTCTCCCTCTTTATGCCTTAGTTACACAGATGGGTTCATCAATG
SlMLO1_MT GATCATACAGGTCCATTGCAGCTATGTGACTCTCCCTCTTTATGCCTTAGTTACACAGATGGGTTCATCAATG
SlMLO1_MT_ AK322443 GATCATACAGGTCCATTGCAGCTATGTGACTCTCCCTCTTTATGCCTTAGTTACACAGATGGGTTCATCAATG
SlMLO1_Heinz GATCATACAGGTCCATTGCAGCTATGTGACTCTCCCTCTTTATGCCTTAGTTACACAGATGGGTTCATCAATG
SlMLO1_MM GATCATACAGGTCCATTGCAGCTATGTGACTCTCCCTCTTTATGCCTTAGTTACACAGATGGGTTCATCAATG
1,390 1,400 1,410 1,420 1,430 1,440 1,450
SlMLO1_M200 AAGCCTATCATCTTTGGTGATAATGTGGCAACAGCTCTTAGAAGCTGGCACCATACAGCGAAAAAACGGGTGA
SlMLO1_MT AAGCCTATCATCTTTGGTGATAATGTGGCAACAGCTCTTAGAAGCTGGCACCATACAGCGAAAAAACGGGTGA
SlMLO1_MT_ AK322443 AAGCCTATCATCTTTGGTGATAATGTGGCAACAGCTCTTAGAAGCTGGCACCATACAGCGAAAAAACGGGTGA
SlMLO1_Heinz AAGCCTATCATCTTTGGTGATAATGTGGCAACAGCTCTTAGAAGCTGGCACCATACAGCGAAAAAACGGGTGA
SlMLO1_MM AAGCCTATCATCTTTGGTGATAATGTGGCAACAGCTCTTAGAAGCTGGCACCATACAGCGAAAAAACGGGTGA

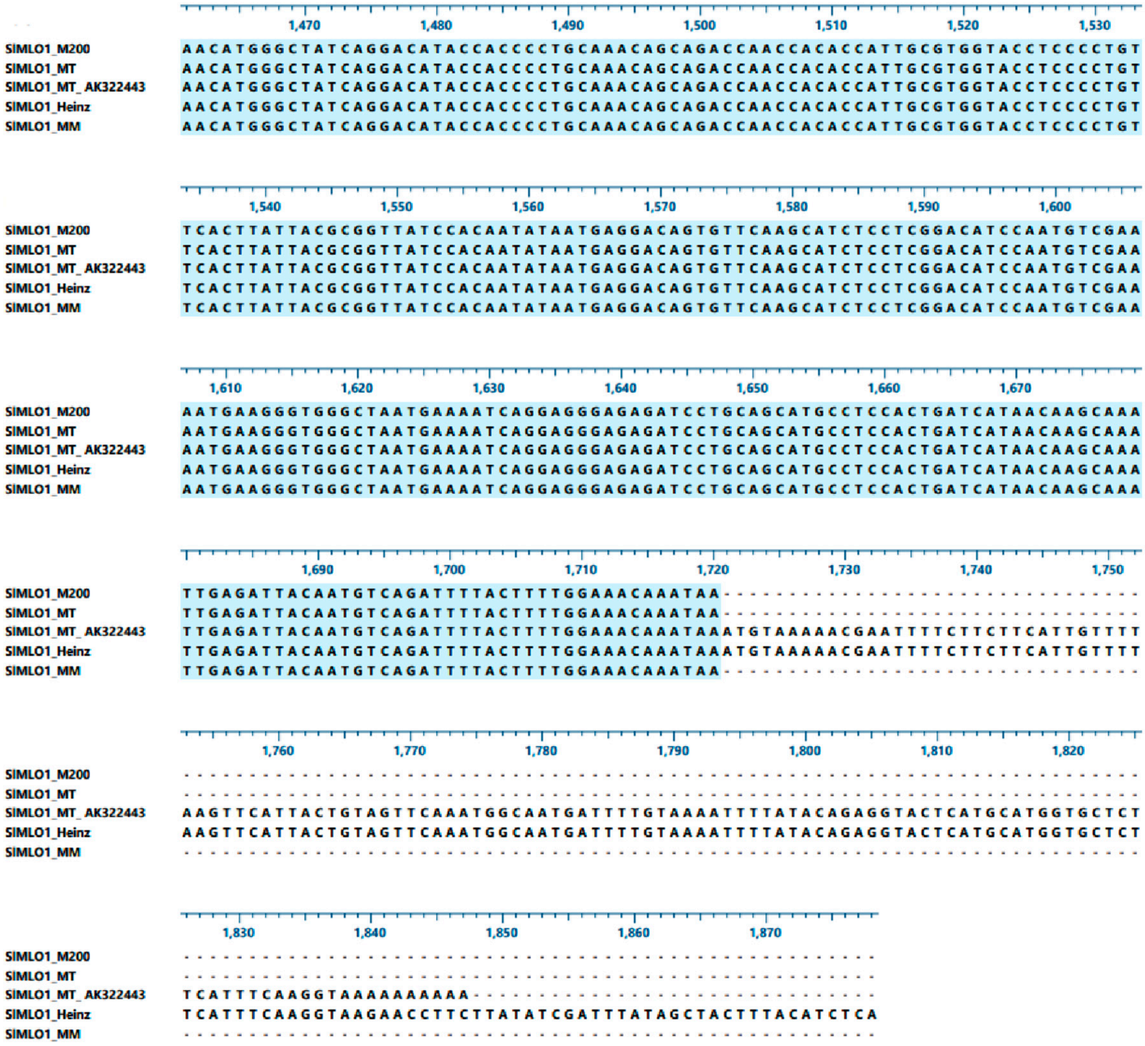

Figure S1. Nucleotide alignment of the *SlMLO1* sequence experimentally obtained from the tomato cv Micro-Tom (MT), the full-length transcript AK322443 of MT *SlMLO1* obtained from NCBI, the *SlMLO1* gene in M200, MM leaf cDNA sequence of *SlMLO1* obtained from Zheng et al. [22] and the one from the cv Heinz as in the SGN database Solyc04g49090.3). Arrow indicates the base change T → A responsible for the premature stop codon in M200 plants.

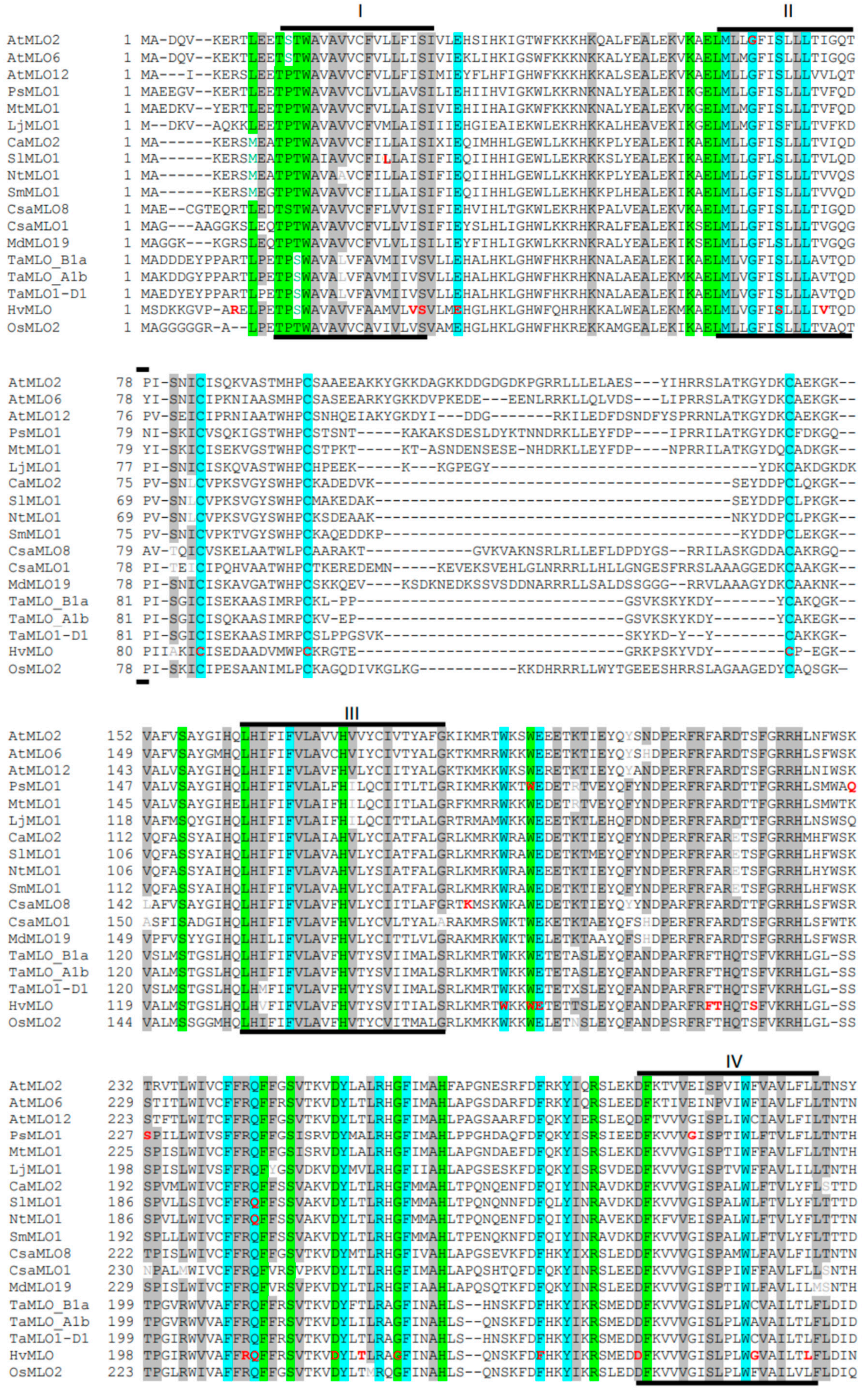
I
II
III
IV

```
                 V                                                              VI
AtMLO2      312 GLRSYLWLPFIPLVVILIVGTKLEVIITKLGLRIQEKGDVVRGAPVVQPGDDLFWFGKPRFILFLIHLVLFTNAFQLAFF
AtMLO6      309 GLNSYLWLPFIPFIVILIVGTKLQVIITKLGLRIQEKGDVVKGTPLVQPGDHFFWFGRPRFILFLIHLVLFTNAFQLAFF
AtMLO12     303 GWDSYLWLPFLPLIVILIVGAKLQMIISKLGLRIQEKGDVVKGAPVVEPGDDLFWFGRPRFILFLIHLVLFTNAFQLAFF
PsMLO1      307 GWYSYYWLPFLPLIVILLVGAKLQMIITKMGLRIQDRGEVIKGAPVVEPGDHLFWFNRPHLLLFTIHLVLFQNAFQLAFF
MtMLO1      305 GWYSSYWLPFLPLIIILLVGAKLQMIITKMGLRIQDRGEVIKGAPVVEPGDHLFWFNSPNLLLFIIHLVLFQNAFQLAFF
LjMLO1      278 GWHSYLWLPFIPLIIILLVGTKLQMIITNMGLKIQERGDVIKGAPLVEPGDDLFWFNRPRLILSLVHLVLFQNAFQLAFF
CaMLO2      272 GVYSYLWVPFVPLIIILLVGTKLQMIITEMGVRISERGDIVKGVPVVEIGDHLFWFNRPGLVLFFINFVLFQNAFQVAFF
SlMLO1      266 RLYSYLWVPFIPLVIILLVGTKLQMIITEMGVRISERGDIVKGVPVVETGDHLFWFNRPALVLFLINFVLFQNAFQVAFF
NtMLO1      266 GLYSYLWVPFIPLVIILLVGTKLEMIIAEMGVRISKRGDIVRGVPVVETGDHLFWFNRPGFVLFLINFVLFQNAFQVAFF
SmMLO1      272 GLYSYLWVPFVPLIIILLVGTKLQMIITEMGVRISERGDIVKGVPVVETGDHLFWFNRPGLVLFLINFVLFQNAFQVAFF
CsaMLO8     302 GWYSYLWLPFISLIIILLVGTKLHVIITHMGLTIQERGHVVKGVPVVQPRDDLFWFGRPQLILFLIHFVLFMNAFQLAFF
CsaMLO1     310 GWRAYLWLPFIPLIILLIGTKLQVIITKMALRIQERGEVVKGVPVVEPGDDLFWFNRPRLILYLINFVLFQNAFQVAFF
MdMLO19     309 GSRSYLWLPFVPLVMILMVGTKLQVIITKMGLKLSERGEVVRGTPLVEPGDHLFWFNNPRLLLYIIHFVLFQNAFALAFF
TaMLO_B1a   277 GIGTLTWISFIPLVILLCVGTKLEMIIMEMALEIQDRASVIKGAPVVEPSNKFFWFHRPDWVLFFIHLTLFQNAFQMAHF
TaMLO_A1b   277 GIGTLTWVSFIPLIILLCVGTKLEMIIMEMALEIQDRSSVIKGAPVVEPSNKFFWFHRPDWVLFFIHLTLFQNAFQMAHF
TaMLO_D1    277 GIGTLTWISFIPLVILLCVGTKLEMIIMEMALEIQDRASVIKGAPVVEPSNKFFWFHRPDWVLFFIHLTLFQNAFQMAHF
HvMLO       276 GVGTLIWISFIPLVILLCVGTKLEMIIMEMALEIQDRASVIKGAPVVEPSNKFFWFHRPDWVLFFIHLTLFQNAFQMAHF
OsMLO2      301 GFGTLIWISFVPLVILMLVGTKLEMVIMEMAQEIQDRATVIKGAPVVEPSNKYFWFNRPDWVLFFIHLILFQNAFQMAHF

                                                             VII
AtMLO2      392 AWST----------YE--FNLNNCFHESTADVVIRLVVGAVVQILCSYVTLPLYALVTQMGSKMKPTVFNDRVATALKKW
AtMLO6      389 VWST----------YE--FGLKNCFHESRVDVIIRISIGLLVQILCSYVTLPLYALVTQMGSKMKPTVFNERVATALKSW
AtMLO12     383 VWST----------YE--FTLKNCFHHKTEDIAIRITMGVLIQVLCSYITLPLYALVTQMGTSMRPTIFNDRVANALKKW
PsMLO1      387 AWST----------YE--FSITSCFHKTTADSVIRITVGVVIQTLCSYVTLPLYALVTQMGSTMKPTIFNERVATALKNW
MtMLO1      385 SWST----------YE--FSINSCFHRTTADNVIRVSVGILIQFLCSYVTLPLYALVTQMGSTMKPTIFNERLATALKKW
LjMLO1      358 AWSA----------CDNDFKINSCFHRSTADVVIRLTLGVVTQVLCSYVTLPLYALVTQMGSTMRPTIFHDRVATALKSW
CaMLO2      352 VWSW----------WK--FGFPSCFHRNAADLAIRLTMGVIIQVHCSYVTLPLYALVTQMGSSMKPIIFGDNVATALRSW
SlMLO1      346 FWSW----------WK--FGFPSCFHKNAADLAIRLTMGVIIQVHCSYVTLPLYALVTQMGSSMKPIIFGDNVATALRSW
NtMLO1      346 VWSW----------WK--FSYPSCFHQNAADIAIRLTMGVIIQVHCSYVTLPLYALVTQMGTSMKPIIFGDNVATALRSW
SmMLO1      352 VWSW----------WK--FDFPSCFHKNAADLAIRLTMGVIIQVHCSYVTLPLYALVTQMGSSMKPIIFGDNVATALRSW
CsaMLO8     324 AWTTYA------------FKWMGCFHQRVEDIVIRLSMGVIIQVLCSYVTLPLYALVTQMGSNMRPTIFNDRVATALKNW
CsaMLO1     390 AWTWY------------EFGLNSCFHEHIEDVVIRISMGVLVQILCSYVTLPLYALVTQMGSTMKPTIFNERVAEALRNW
MdMLO19     389 AWTWSGKGGLHFTLLQYEFGLKSCFHEKLEDVVLRISMGVIIQILCSYVTLPLYALVTQMGSTMKPVIFNDRVATALKKW
TaMLO_B1a   357 VWTV----------AT--PGLKKCFHMHIGLSIMKVVLGLALQFLCSYITFPLYALVTQMGSNMKRSIFDEQTAKALTNW
TaMLO_A1b   357 VWTV----------AT--PGLKDCFHMNIGLSIMKVVLGLALQFLCSYITFPLYALVTQMGSNMKRSIFDEQTAKALTNW
TaMLO_D1    357 VWTV----------AT--PGLKKCFHMHIGLSIMKVVLGLALQFLCSYITFPLYALVTQMGSNMKRSIFDEQTAKALTNW
HvMLO       356 VWTV----------AT--PGLKKCYHTQIGLSIMKVVLGLALQFLCSYMTFPLYALVTQMGSNMKRSIFDEQTSKALTNW
OsMLO2      381 VWTL----------AT--PGLKKCFHENMGLSIMKVVVGIFIQFLCSYSTFPLYALVTQMGSNMKKTIFEEQTMKALMNW

                CaMBD
AtMLO2      460 HHTAKNETKHGRHSG-----S--NTPFSSRPTTPTHGSSPIHLLHNF---NNRSV-E--NYPSSPSPR----YSGHG---
AtMLO6      457 HHTAKKNIKHGRTSE-----S--TTPFSSRPTTPTHGSSPIHLLRNAPHKRSRSVDE--SFANSFSPR----NS------
AtMLO12     451 HHTAKKQTKHG-HSG-----S--NTPHSSRPTTPTHGMSPVHLLHNY---NNRSLDQQTSFTASPSPPRFSDYSGQG---
PsMLO1      455 HHTAKKQVKQSNHSN-----N--TTPYSSRPSTPTHAMSPVHLLHRH-TAGN-------SDSLQTSPE-----KS-----
MtMLO1      453 HHTAKKQVKHNKHSN-----N--TTPYSSRQSTPTHGMSPVHLLHRQ-TFGN-------SDSLQTSPR-----TS-----
LjMLO1      428 HHTAKKHVKHNRDSN-----SHSNTPFSSRPATPTHGMSPVHLLHKHHNYHN-------SDSPLASPR-----ESPS---
CaMLO2      420 HNTAKKRVRHGRVSE-----N--TTPISSRPATPLRGTSPVHLLRGYPKY-N-------EDNVQAYPRT-------S---
SlMLO1      414 HHTAKKRVKHG-LSG-----H--TTPANSRPTTPLRGTSPVHLLRGYPQY-N-------EDSVQASPRT-------S---
NtMLO1      414 HNTAKKRVKHGRLSE-----N--TTPVSSRPATPLHGTSPVHLLRSYPQYSN-------EES-----RT-------S---
SmMLO1      420 HHMAKKRVKHGRLS-------GNTTPVSSRPTTPLHGTSPVHLLRGYPQYN--------EDSVQASPRT-------S---
CsaMLO8     392 HHSAKKNMKQHRNPD-------STSPFSSRPATPTHGMSPIHLLHKH-------------QHGSTSPR------------
CsaMLO1     458 YHSARKHIKHNRGS---------VTPMSSRPATPTHSMSPVHLLRHY---------KSEVDSFHTSPRRSPFDTDRWDND
MdMLO19     469 HIAAKKHVKHKNAS-----------P-ASAPGTPLHSMSPVHLLRNYKYEQDI-------DSIQTSPRMPYFDNEGSDSP
TaMLO_B1a   425 RNTAKEKKKVRDTDMLMAQMIGDATPSRGASPMPSRGSSPVHLLHK---------GMGRSDDPQSTPT------------
TaMLO_A1b   425 RNTAKEKKKVRDTDMLMAQMIGDATPSRGTSPMPSRGSSPVHLLQK---------GMGRSDDPQSAPT------------
TaMLO_D1    425 RNTAKEKKKVRDTDMLMAQMIGDATPSRGTSPMPSRASSPVHLLHK---------GMGRSDDPQSAPTS----------
HvMLO       424 RNTAKEKKKVRDTDMLMAQMIGDATPSRGSSPMPSRGSSPVHLLHK---------GMGRSDDPQSAPT------------
OsMLO2      449 RKTAREKKKLRDADEFLAQMSGDTTPSRGS--------SPVHLLHK---------QRVRSEDPPSAPA------------

AtMLO2      520 --HHEHQFWDPESQHQE-------AETST--H-HS-LAHESSEP--VLASVELPP----IRTSK-----SLRDFSFKK--
AtMLO6      518 ----DFDSWDPESQHET-------AETSNSNH-RSRFGEEESEKKFVSSSVELPPGPGQIRTQHEISTISLRDFSFKR--
AtMLO12     517 --HGHQHFFDPESQNHSYQREITDSEFSNSHHPQVDMASPVREEKEIVEHVKV----------------DLSEFTFKK--
PsMLO1      510 --DYKNEQWDIEGE----------GPTSLRNDQT---GQHE-IQIAGVESFSSTELPVRIR-HE--STSGSKDFSFEKRH
MtMLO1      508 --NYENEQWDVEGG----------GSTSPRNNQT---VASE-IEIPIVESFSTTELPVSVR-HEIGTTSSSKDFSFEKRH
LjMLO1      488 --NYETEQWYLE-------------PNSPSNHTR---GHDQTLQMQVLGSSATEFSPAEVH-HEI-TPIGLPEFSFDKAP
CaMLO2      475 --NVENEGWANETS-------------TENK--------DHQEEGQILQHASTSMQHPHTDQHQI--EIAMSDFTFGNK-
SlMLO1      468 --NVENEGWANEN-----------------------------QEGEILQHASTD--H----NKQI--EITMSDFTFGNK-
NtMLO1      465 --NAENEGWANEIP-------------TSPRRQIENIKDDDHQEGEI--HASSSV-------HQV--EIAMSEFTFGNK-
SmMLO1      475 --NVENEGWANEIS----------------------TDNKDYQEGHASTSVRPP------HAHNQQIEITMSDFTFGNK-
CsaMLO8     440 LSDAEPDRWEELPPSSHHSRAPHHDNHQDQQEQSETIIREQEMTVQGPSSSETGSITRPARPHQEITRTP-SDFSFAKX-
CsaMLO1     520 ---------SPSPSRH--------VDGSSSSQPHVEMGGYEKDPVESSSSQVDPVQPSRNRNQHEIHIGGPKDFSFDR--
MdMLO19     530 FHHQDNLTWSQQGTN----------------------MEGQKEEISAHGPNAESNALGAYGSIIQHEIQIHSAALTFEK--
TaMLO_B1a   484 ---------SPRAMEEARDMYPVVVA-----HPVHRLNPADRRRSVSSSALDVD--------------IPSADFSFS-Q-
TaMLO_A1b   484 ---------SPRTMEEARDMYPVVVA-----HPVHRLNPADRRRSVSSSALDAD--------------IPSADFSFS-Q-
TaMLO_D1    485 ----------PRTMEEARDMYPVVVA-----HPVHRLNPADRRRSVSSSALDAD--------------IPSADFSFS-Q-
HvMLO       483 ---------SPRTQQEARDMYPVVVA-----HPVHRLNPNDRRRSASSSALEAD--------------IPSADFSFS-Q-
OsMLO2      500 ---------SPGFAGEARDMYPVPVAPVVRPHGFNRTDP-DKRRAASSSAIQVD--------------IADSDFSFSVQ-
```

```
AtMLO2      573 ------------------------K------
AtMLO6      583 ------------------------R------
AtMLO12     576 ------------------------K------
PsMLO1      568 ------------------------KRHLGSN
MtMLO1      568 ------------------------KRHIGSN
LjMLO1      545 ------------------------KAPTSRE
CaMLO2      527 ------------------------NK---X-
CsaMLO8     516 ------------------------KX-----
CsaMLO1     580 ------------------------R---VEX
SlMLO1      506 ------------------------NK---X-
NtMLO1      516 ------------------------NK---MS
SmMLO1      522 ------------------------NK-----
MdMLO19     586 ------------------------K--TERS
TaMLO_B1a   533 -------------------------Q----G
TaMLO_A1b   533 -------------------------Q----G
TaMLO1-D1   534 GXDKFLYXCXSNVXPTXDVMIRTIRNTNFYX
HvMLO       532 -------------------------Q----G
OsMLO2      553 ------------------------VQ----R
```

Figure S2. Protein alignment of functionally characterized MLO sequences of *Arabidopsis* thaliana AtMLO2, -6, and -12 (GenBank accession numbers NP172598, NP176350, and NP565902) [17], *Pisum sativum* (pea) PsMLO1 (GenBank accession number FJ463618) [1], *Medicago truncatula* (barrel clover) MtMLO1 (GenBank accession number HQ446457) [1], *Lotus japonicus* LjMLO1 (GenBank accession number AY967410) [1], *Capsicum annuum* (pepper) CaMLO2 (GenBank accession number AFH68055) [23], *Cucumis sativus* (cucumber) CsaMLO1 and -8 (GenBank accession numbers Csa1M085890.1 and Csa5M623470.1) [12,14], *Solanum lycopersicum* (tomato) SlMLO1 (GenBank accession number NP001234814) [7], *Nicotiana tabacum* (tobacco) NtMLO1 (GenBank accession number KM244716) [15], *S. melongena* (eggplant) SmMLO1 (GenBank accession number KM244717) [24], *Malus domestica* (apple) MdMLO19 (GenBank accession number MDP0000168714) [10], *Triticum aestivum* (wheat) TaMLO-A1b, TaMLO-B1a, and TaMLO-D1 (GenBank accession numbers AX063298, AF361932, and AX063296) [25,26], *Hordeum vulgare* (barley) HvMLO (GenBank accession number Z83834) [27] and *Oryza sativa* (rice) OsMLO2 (GenBank accession number AF384030) [25]. Highlighted in green and in light blue are the conserved amino acids among the whole MLO family indicated by Kusch et al. [28] and by Elliott et al. [29], respectively. Amino acids highlighted in gray refer to the ones reported to be under negative selection by Appiano et al. [30]. Letters displayed in green, light blue or gray indicate synonymous amino acid exchanges in each of three categories above described. Letters in red bold indicate amino acids identified in *mlo*-mutants for each of the plant species described above. Black lines indicate the position of the transmembrane domains which have been numbered with romans numbers.

References

1. Humphry, M.; Reinstädler, A.; Ivanov, S.; Bisseling, T.; Panstruga, R. Durable broad-spectrum powdery mildew resistance in pea er1 plants is conferred by natural loss-of-function mutations in PsMLO1. *Mol. Plant Pathol.* **2011**, *12*, 866–878, doi:10.1111/j.1364-3703.2011.00718.x.
2. Pavan, S.; Schiavulli, A.; Appiano, M.; Marcotrigiano, A.R.; Cillo, F.; Visser, R.G.F.; Bai, Y.; Lotti, C.; Ricciardi, L. Pea powdery mildew *er1* resistance is associated to loss-of-function mutations at a *MLO* homologous locus. *Theor. Appl. Genet.* **2011**, *123*, 1425–1431, doi:10.1007/s00122-011-1677-6.
3. Sun, S.; Fu, H.; Wang, Z.; Duan, C.; Zong, X.; Zhu, Z. Discovery of a Novel *er1* Allele Conferring Powdery Mildew Resistance in Chinese Pea (*Pisum sativum* L.) Landraces. *PLoS ONE* **2016**, *11*, e0147624, doi:10.1371/journal.pone.0147624.
4. Santo, T.; Rashkova, M.; Alabaça, C.; Leitão, J. The ENU-induced powdery mildew resistant mutant pea (*Pisum sativum* L.) lines S(*er1mut1*) and F(*er1mut2*) harbour early stop codons in the *PsMLO1* gene. *Mol. Breed.* **2013**, *32*, 723–727, doi:10.1007/s11032-013-9889-x.
5. Jiang, P.; Chen, Y.; Wilde, H.D. Identification and mutagenesis of disease susceptibility genes of *Petunia hybrida*. *Plant Cell, Tissue Organ. Cult.* **2016**, *126*, 117–125, doi:10.1007/s11240-016-0982-9.
6. Kaufmann, H.; Qiu, X.; Wehmeyer, J.; Debener, T. Isolation, Molecular Characterization, and Mapping of Four Rose *MLO* Orthologs. *Front. Plant Sci.* **2012**, *3*, 244, doi:10.3389/fpls.2012.00244.
7. Bai, Y.; Pavan, S.; Zheng, Z.; Zappel, N.F.; Reinstädler, A.; Lotti, C.; De Giovanni, C.; Ricciardi, L.; Lindhout, P.; Visser, R.; et al. Naturally Occurring Broad-Spectrum Powdery Mildew Resistance in a Central American Tomato Accession Is Caused by Loss of *Mlo* Function. *Mol. Plant Microbe Interac.* **2008**, *21*, 30–39, doi:10.1094/mpmi-21-1-0030.
8. Kim, D.; Jin, B.; Je, B.I.; Choi, Y.; Kim, B.S.; Jung, H.J.; Nou, I.S.; Park, Y. Development of DNA markers for *Slmlo1.1*, a new mutant allele of the powdery mildew resistance gene *SlMlo1* in tomato (*Solanum lycopersicum*). *Genome* **2018**, *61*, 703-12, doi: 10.1139/gen-2018-0114
9. Nekrasov, V.; Wang, C.; Win, J.; Lanz, C.; Weigel, D.; Kamoun, S. Rapid generation of a transgene-free powdery mildew resistant tomato by genome deletion. *Sci. Rep.* **2017**, *7*, 1–6, doi:10.1038/s41598-017-00578-x.
10. Pessina, S.; Angeli, D.; Martens, S.; Visser, R.G.; Bai, Y.; Salamini, F.; Velasco, R.; Schouten, H.J.; Malnoy, M. The knock-down of the expression of *MdMLO19* reduces susceptibility to powdery mildew (*Podosphaera leucotricha*) in apple (*Malus domestica*). *Plant Biotechnol. J.* **2016**, *14*, 2033–2044, doi:10.1111/pbi.12562.

11. Wan, D.-Y.; Guo, Y.; Cheng, Y.; Hu, Y.; Xiao, S.; Wang, Y.; Wen, Y.-Q. CRISPR/Cas9-mediated mutagenesis of *VvMLO3* results in enhanced resistance to powdery mildew in grapevine (*Vitis vinifera*). *Hortic. Res.* **2020**, *7*, 1–14, doi:10.1038/s41438-020-0339-8.
12. Berg, J.A.; Appiano, M.; Bijsterbosch, G.; Visser, R.G.F.; Schouten, H.J.; Bai, Y. Functional characterization of cucumber (*Cucumis sativus* L.) Clade V *MLO* genes. *BMC Plant Biol.* **2017**, *17*, 1-15, doi: 10.1186/s12870-017-1029-z
13. Nie, J.; Wang, Y.; He, H.; Guo, C.; Zhu, W.; Pan, J.; Li, D.; Lian, H.; Pan, J.; Cai, R. Loss-of-function mutations in *CsMLO1* confer durable powdery mildew resistance in cucumber (*Cucumis sativus* L.). *Front. Plant Sci.* **2015**, *6*, 1155. doi.org/10.3389/fpls.2015.01155
14. Berg, J.A.; Appiano, M.; Martínez, M.S.; Hermans, F.W.K.; Vriezen, W.H.; Visser, R.G.F.; Bai, Y.; Schouten, H.J. A transposable element insertion in the susceptibility gene *CsaMLO8* results in hypocotyl resistance to powdery mildew in cucumber. *BMC Plant Biol.* **2015**, *15*, 243, doi:10.1186/s12870-015-0635-x.
15. Appiano, M.; Pavan, S.; Catalano, D.; Zheng, Z.; Bracuto, V.; Lotti, C.; Visser, R.G.F.; Ricciardi, L.; Bai, Y. Identification of candidate *MLO* powdery mildew susceptibility genes in cultivated Solanaceae and functional characterization of tobacco *NtMLO1*. *Transgenic Res.* **2015**, *24*, 847-858, doi: 10.1007/s11248-015-9878-4
16. Fujimura, T.; Sato, S.; Tajima, T.; Arai, M. Powdery mildew resistance in the Japanese domestic tobacco cultivar Kokubu is associated with aberrant splicing of *MLO* orthologues. *Plant Pathol.* **2016**, *65*, 1358–1365, doi:10.1111/ppa.12498.
17. Consonni, C.; Humphry, M.E.; Hartmann, H.A.; Livaja, M.; Durner, J.; Westphal, L.; Vogel, J.; Lipka, V.; Kemmerling, B.; Schulze-Lefert, P.; et al. Conserved requirement for a plant host cell protein in powdery mildew pathogenesis. *Nat. Genet.* **2006**, *38*, 716–720, doi:10.1038/ng1806.
18. Reinstädler, A.; Müller, J.; Czembor, J.H.; Piffanelli, P.; Panstruga, R. Novel induced *mlo* mutant alleles in combination with site-directed mutagenesis reveal functionally important domains in the heptahelical barley Mlo protein. *BMC Plant Biol.* **2010**, *10*, 31, doi:10.1186/1471-2229-10-31.
19. Ge, X.; Deng, W.; Lee, Z.Z.; Lopez-Ruiz, F.J.; Schweizer, P.; Ellwood, S.R. Tempered *mlo* broad-spectrum resistance to barley powdery mildew in an Ethiopian landrace. *Sci. Rep.* **2016**, *6*, 1-10, doi: 10.1038/srep29558
20. Acevedo-Garcia, J.; Spencer, D.; Thieron, H.; Reinstädler, A.; Hammond-Kosack, K.; Phillips, A.L.; Panstruga, R. *mlo*-based powdery mildew resistance in hexaploid bread wheat generated by a non-transgenic TILLING approach. *Plant Biotechnol. J.* **2016**, *15*, 367–378, doi:10.1111/pbi.12631.
21. Wang, Y.; Cheng, X.; Shan, Q.; Zhang, Y.; Liu, J.; Gao, C.; Qiu, J.-L. Simultaneous editing of three homoeoalleles in hexaploid bread wheat confers heritable resistance to powdery mildew. *Nat. Biotechnol.* **2014**, *32*, 947–951, doi:10.1038/nbt.2969.
22. Zheng, Z.; Appiano, M.; Pavan, S.; Bracuto, V.; Ricciardi, L.; Visser, R.G.F.; Wolters, A.-M.A.; Bai, Y. Genome-Wide Study of the Tomato *SlMLO* Gene Family and Its Functional Characterization in Response to the Powdery Mildew Fungus *Oidium neolycopersici*. *Front. Plant Sci.* **2016**, *7*, 380, doi:10.3389/fpls.2016.00380.
23. Zheng, Z.; Nonomura, T.; Appiano, M.; Pavan, S.; Matsuda, Y.; Toyoda, H.; Wolters, A.M.A.; Visser, R.G.F.; Bai, Y. Loss of function in *Mlo* orthologs reduces susceptibility of pepper and tomato to powdery mildew disease caused by *Leveillula taurica*. *PLoS ONE* **2013**, *8*, e70723, doi: 10.1371/journal.pone.0070723
24. Bracuto, V.; Appiano, M.; Ricciardi, L.; Göl, D.; Visser, R.G.F.; Bai, Y.; Pavan, S. Functional characterization of the powdery mildew susceptibility gene *SmMLO1* in eggplant (*Solanum melongena* L.). *Transgenic Res.* **2017**, *26*, 323-330, doi: 10.1007/s11248-016-0007-9
25. Elliott, C.; Zhou, F.; Spielmeyer, W.; Panstruga, R.; Schulze-Lefert, P. Functional conservation of wheat and rice *Mlo* orthologs in defense modulation to the powdery mildew fungus. *Mol. Plant Microbe Interact.* **2002**, *15*, 1069-1077, doi: 10.1094/MPMI.2002.15.10.1069
26. Várallyay, É.; Giczey, G.; Burgyán, J. Virus-induced gene silencing of *Mlo* genes induces powdery mildew resistance in *Triticum aestivum*. *Arch. Virol.* **2012**, *157*, 1345-1350, doi: 10.1007/s00705-012-1286-y
27. Büschges, R.; Hollricher, K.; Panstruga, R.; Simons, G.; Wolter, M.; Frijters, A.; van Daelen, R.; van der Lee, T.; Diergaarde, P.; Groenendijk, J.; et al. The Barley *Mlo* Gene: A Novel Control Element of Plant Pathogen Resistance. *Cell* **1997**, *88*, 695–705, doi:10.1016/s0092-8674(00)81912-1.
28. Kusch, S.; Pesch, L.; Panstruga, R. Comprehensive Phylogenetic Analysis Sheds Light on the Diversity and Origin of the MLO Family of Integral Membrane Proteins. *Genome Biol. Evol.* **2016**, *8*, 878–895, doi:10.1093/gbe/evw036.
29. Elliott, C.; Müller, J.; Miklis, M.; Bhat, R.A.; Schulze-Lefert, P.; Panstruga, R. Conserved extracellular cysteine residues and cytoplasmic loop–loop interplay are required for functionality of the heptahelical MLO protein. *Biochem. J.* **2005**, *385*, 243–254, doi:10.1042/bj20040993.
30. Appiano, M.; Catalano, D.; Martínez, M.S.; Lotti, C.; Zheng, Z.; Visser, R.G.F.; Ricciardi, L.; Bai, Y.; Pavan, S. Monocot and dicot MLO powdery mildew susceptibility factors are functionally conserved in spite of the evolution of class-specific molecular features. *BMC Plant Biol.* **2015**, *15*, 1–10, doi:10.1186/s12870-015-0639-6.
